# Chaos as an intermittently forced linear system

**DOI:** 10.1038/s41467-017-00030-8

**Published:** 2017-05-30

**Authors:** Steven L. Brunton, Bingni W. Brunton, Joshua L. Proctor, Eurika Kaiser, J. Nathan Kutz

**Affiliations:** 1 Department of Mechanical Engineering, University of Washington, Seattle, WA 98195 USA; 2 Department of Biology, University of Washington, Seattle, WA 98195 USA; 3 Institute for Disease Modeling, Bellevue, WA 98004 USA; 4 Department of Applied Mathematics, University of Washington, Seattle, WA 98195 USA

## Abstract

Understanding the interplay of order and disorder in chaos is a central challenge in modern quantitative science. Approximate linear representations of nonlinear dynamics have long been sought, driving considerable interest in Koopman theory. We present a universal, data-driven decomposition of chaos as an intermittently forced linear system. This work combines delay embedding and Koopman theory to decompose chaotic dynamics into a linear model in the leading delay coordinates with forcing by low-energy delay coordinates; this is called the Hankel alternative view of Koopman (HAVOK) analysis. This analysis is applied to the Lorenz system and real-world examples including Earth’s magnetic field reversal and measles outbreaks. In each case, forcing statistics are non-Gaussian, with long tails corresponding to rare intermittent forcing that precedes switching and bursting phenomena. The forcing activity demarcates coherent phase space regions where the dynamics are approximately linear from those that are strongly nonlinear.

## Abstract (web summary)

The huge amount of data generated in fields like neuroscience or finance calls for effective strategies that mine data to reveal underlying dynamics. Here Brunton* et al.*develop a data-driven technique to analyze chaotic systems and predict their dynamics in terms of a forced linear model.

## Introduction

Dynamical systems describe the changing world around us, modeling the interactions between quantities that co-evolve in time^1^. These dynamics often give rise to rich, complex behaviors that may be difficult to predict from uncertain measurements, a phenomena commonly known as chaos. Chaotic dynamics are ubiquitous in the physical, biological, and engineering sciences, and they have captivated amateurs and experts for over a century. The motion of planets^2^, weather and climate^3^, population dynamics^4-6^, epidemiology^7^, financial markets, earthquakes, and turbulence^8, 9^, are all compelling examples of chaos. Despite the name, chaos is not random, but is instead highly organized, exhibiting coherent structure and patterns^10, 11^.

The confluence of big data and machine learning is driving a paradigm shift in the analysis and understanding of dynamical systems in science and engineering. Data are abundant, while physical laws or governing equations remain elusive, as is true for problems in climate science, finance, and neuroscience. Even in classical fields such as turbulence, where governing equations do exist, researchers are increasingly turning toward data-driven analysis^12-16^. Many critical data-driven problems, such as predicting climate change, understanding cognition from neural recordings, or controlling turbulence for energy efficient power production and transportation, are primed to take advantage of progress in the data-driven discovery of dynamics^17–27^.

An early success of data-driven dynamical systems is the celebrated Takens embedding theorem^9^, which allows for the reconstruction of an attractor that is diffeomorphic to the original chaotic attractor from a time series of a single measurement. This remarkable result states that, under certain conditions, the full dynamics of a system as complicated as a turbulent fluid may be uncovered from a time series of a single point measurement. Delay embeddings have been widely used to analyze and characterize chaotic systems^5–7, 28–31^. They have also been used for linear system identification with the eigensystem realization algorithm (ERA)^32^ and in climate science with the singular spectrum analysis (SSA)^33^ and nonlinear Laplacian spectrum analysis^34^. ERA and SSA yield eigen-time-delay coordinates by applying principal component analysis to a Hankel matrix. However, these methods are not generally useful for identifying meaningful models of chaotic nonlinear systems, such as those considered here.

In this work, we develop a universal data-driven decomposition of chaos into a forced linear system. This decomposition relies on time-delay embedding, a cornerstone of dynamical systems, but takes a new perspective based on regression models^19^ and modern Koopman operator theory^35–37^. The resulting method partitions phase space into coherent regions where the forcing is small and dynamics are approximately linear, and regions where the forcing is large. The forcing may be measured from time series data and strongly correlates with attractor switching and bursting phenomena in real-world examples. Linear representations of strongly nonlinear dynamics, enabled by machine learning and Koopman theory, promise to transform our ability to estimate, predict, and control complex systems in many diverse fields. A video abstract is available for this work at: https://youtu.be/831Ell3QNck, and code is available at: http://faculty.washington.edu/sbrunton/HAVOK.zip.

## Results

### Linear representations of nonlinear dynamics

Consider a dynamical system^1^ of the form

1 $$\frac{\rm{d}}{{{\rm d}t}}{\bf{x}}(t) = {\bf{f}}({\bf{x}}(t)),$$

where ${\bf{x}}(t) \in {{\Bbb R}^n}$ is the state of the system at time *t* and **f** represents the dynamic constraints that define the equations of motion. When working with data, we often sample (1) discretely in time:

2 $${{\bf{x}}_{k + 1}} = {\bf{F}}({{\bf{x}}_k}) = {{\bf{x}}_k} + {\int}_{k\Delta t}^{(k + 1)\Delta t} {\bf{f}}({\bf{x}}(\tau ))\rm{d}\tau ,$$

where **x**_k_ = **x**(*k*Δ*t*). The traditional geometric perspective of dynamical systems describes the topological organization of trajectories of (1) or (2), which are mediated by fixed points, periodic orbits, and attractors of the dynamics **f**. However, analyzing the evolution of measurements, *y* = *g*(**x**), of the state provides an alternative view. This perspective was introduced by Koopman in 1931^38^, although it has gained traction recently with the pioneering work of Mezic *et al.* ^35, 36^ in response to the growing abundance of measurement data and the lack of known governing equations for many systems of interest. Koopman analysis relies on the existence of a linear operator ${\cal K}$ for the dynamical system in (2), given by

3 $${\cal K}g \buildrel \Delta \over = g \circ {\bf{F}}\quad \Rightarrow \quad {\cal K}g({{\bf{x}}_k}) = g({{\bf{x}}_{k + 1}}).$$

The Koopman operator ${\cal K}$ induces a linear system on the space of all measurement functions *g*, trading finite-dimensional nonlinear dynamics in (2) for infinite-dimensional linear dynamics in (3).

Expressing nonlinear dynamics in a linear framework is appealing because of the wealth of optimal control techniques for linear systems and the ability to analytically predict the future. However, obtaining a finite-dimensional approximation of the Koopman operator is challenging in practice^39^, relying on intrinsic measurements related to the eigenfunctions of the Koopman operator ${\cal K}$, which may be more difficult to obtain than the solution of the original system (2).

### Hankel alternative view of Koopman (HAVOK) analysis

Obtaining linear representations for strongly nonlinear systems has the potential to revolutionize our ability to predict and control these systems. In fact, the linearization of dynamics near fixed points or periodic orbits has long been employed for local linear representation of the dynamics^1^. The Koopman operator is appealing because it provides a global linear representation, valid far away from fixed points and periodic orbits, although previous attempts to obtain finite-dimensional approximations of the Koopman operator have had limited success. Dynamic mode decomposition (DMD)^40–43^ seeks to approximate the Koopman operator with a best-fit linear model advancing spatial measurements from one time to the next. However, DMD is based on linear measurements, which are not rich enough for many nonlinear systems. Augmenting DMD with nonlinear measurements may enrich the model^44^, but there is no guarantee that the resulting models will be closed under the Koopman operator^39^. Details about these related methods are provided in Supplementary Note 2.

Instead of advancing instantaneous measurements of the state of the system, we obtain intrinsic measurement coordinates based on the time-history of the system. This perspective is data-driven, relying on the wealth of information from previous measurements to inform the future. Unlike a linear or weakly nonlinear system, where trajectories may get trapped at fixed points or on periodic orbits, chaotic dynamics are particularly well-suited to this analysis: trajectories evolve to densely fill an attractor, so more data provides more information.

**Fig. 1:**
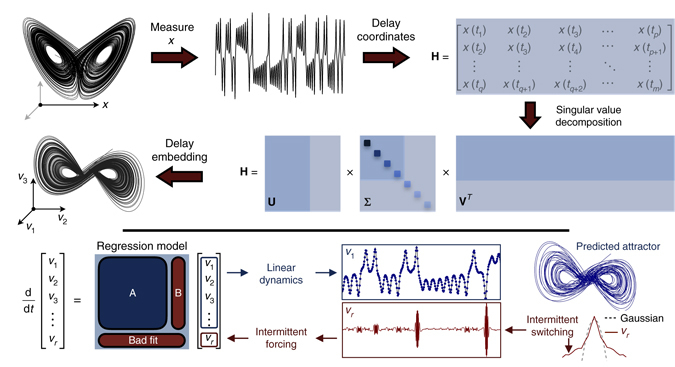
Decomposition of chaos into a linear dynamical system with forcing. A time series *x*(*t*) is stacked into a Hankel matrix **H**. The SVD of **H** yields a hierarchy of eigen time series that produce a delay-embedded attractor. A best-fit linear regression model is obtained on the delay coordinates **v**; the linear fit for the first *r*−1 variables is excellent, but the last coordinate *v*_*r*_ is not well-modeled as linear. Instead, *v*_*r*_(*t*) is a stochastic input that forces the first *r*−1 variables. The rare events in the forcing correspond to lobe switching in the chaotic dynamics. This architecture is called the Hankel alternative view of Koopman (HAVOK) analysis

This method is shown in Fig. 1 for the Lorenz system (details are provided in Supplementary Note 3). The conditions of the Takens embedding theorem are satisfied^9^, so eigen-time-delay coordinates may be obtained from a time series of a single measurement *x*(*t*) by taking a singular value decomposition (SVD) of the following Hankel matrix **H**:

4 $${\bf{H}}{\rm{ = }}\left[ {\begin{array}{*{20}{c}} {x({t_1})} & {x({t_2})} & \cdots & {x({t_p})} \\ \\ {x({t_2})} & {x({t_3})} & \cdots & {x({t_{p + 1}})} \\ \\ \vdots & \vdots & \ddots & \vdots \\ \\ {x({t_q})} & {x({t_{q + 1}})} & \cdots & {x({t_m})} \\ \end{array}} \right]{\rm{ = }}{\bf{U}}\Sigma {{\bf{V}}^{*}}.$$

The columns of **U** and **V** from the SVD are arranged hierarchically by their ability to model the columns and rows of **H**, respectively. Often, **H** may admit a low-rank approximation by the first *r* columns of **U** and **V**. Note that the Hankel matrix in (4) is the basis of ERA^32^ in linear system identification and SSA^33^ in climate time series analysis. Interestingly, a connection between the Koopman operator and the Takens embedding was explored as early as 2004^45^.

The low-rank approximation to (4) provides a data-driven measurement system that is approximately invariant to the Koopman operator for states on the attractor. By definition, the dynamics map the attractor onto itself, making it invariant to the flow. We may re-write (4) with the Koopman operator ${\cal K}$:

5 $${\bf{H}} = \left[ {\begin{array}{*{20}{c}} {x({t_1})} & {{\cal K}x({t_1})} & \cdots & {{{\cal K}^{p - 1}}x({t_1})} \\ \\ {{\cal K}x({t_1})} & {{{\cal K}^2}x({t_1})} & \cdots & {{{\cal K}^p}x({t_1})} \\ \\ \vdots & \vdots & \ddots & \vdots \\ \\ {{{\cal K}^{q - 1}}x({t_1})} & {{{\cal K}^q}x({t_1})} & \cdots & {{{\cal K}^{m - 1}}x({t_1})} \\ \end{array}} \right].$$

The columns of (4), and thus (5), are well-approximated by the first *r* columns of **U**, so these eigen-time-series provide a Koopman-invariant measurement system. The first *r* columns of **V** provide a time series of the magnitude of each of the columns of **U**Σ in the data. By plotting the first three columns of **V**, we obtain an embedded attractor for the Lorenz system, shown in Fig. 1e.

The connection between eigen-time-delay coordinates from (4) and the Koopman operator motivates a linear regression model on the variables in **V**. Even with an approximately Koopman-invariant measurement system, there remain challenges to identifying a linear model for a chaotic system. A linear model, however detailed, cannot capture multiple fixed points or the unpredictable behavior characteristic of chaos with a positive Lyapunov exponent^39^. Instead of constructing a closed linear model for the first *r* variables in **V**, we build a linear model on the first *r*−1 variables and allow the last variable, *v*_*r*_, to act as a forcing term:

6 $$\frac{{\rm d}}{{{\rm d}t}}{\bf{v}}(t) = {\bf{Av}}(t) + {\bf{B}}{v_r}(t).$$

Here $${\bf{v}} = {[ {\begin{array}{*{20}{c}} {{v_1}} & {{v_2}} & \cdots & {{v_{r - 1}}} \end{array}} ]^T}$$ is a vector of the first *r*−1 eigen-time-delay coordinates. In all of the examples below, the linear model on the first *r*−1 terms is accurate, while no linear model represents *v*_*r*_. Instead, *v*_*r*_ is an input forcing to the linear dynamics in (6), which approximate the nonlinear dynamics in (1). The statistics of *v*_*r*_(*t*) are non-Gaussian, as seen in Fig. 1h. The long tails correspond to rare-event forcing that drives lobe switching in the Lorenz system; this is related to rare-event forcing observed and modeled by others^12, 13, 46^. However, the statistics of the forcing alone is insufficient to characterize the switching dynamics, as the timing is crucial. The long-tail forcing comes in high-frequency bursts, which are not captured in the statistics alone. In fact, forcing the system in (6) with other forcing signatures from the same statistics, for example by randomly shuffling the forcing time series, does not result in the same dynamics. Thus, the timing of the forcing is as important as the distribution. In principle, it is also possible to split the variables into *r*−*s* high-energy modes for the linear model and *s* low-energy forcing modes, although this is not explored in the present work. The splitting of dynamics into deterministic linear and chaotic stochastic dynamics was proposed in ref. ^35^. Here we extend this concept to fully chaotic systems where the Koopman operators have continuous spectra and develop a robust numerical algorithm for the splitting.

**Fig. 2:**
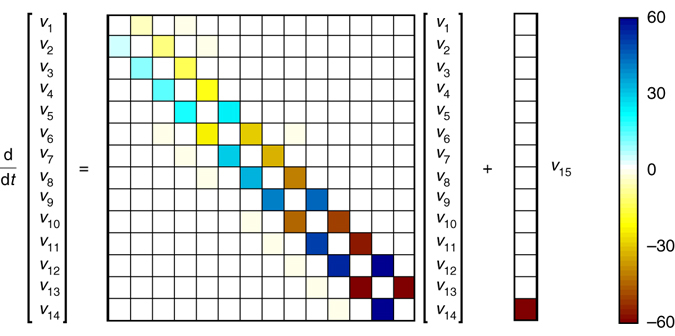
The regression model obtained for the Lorenz system is sparse, having a dominant off-diagonal structure. This HAVOK model is highly structured, with skew symmetric entries that are nearly integer multiples of five; this fascinating structure is explored more in Supplementary Note 4

The forced linear system in (6) was discovered after applying the sparse identification of nonlinear dynamics (SINDy)^19^ algorithm to delay coordinates of the Lorenz system. Even when allowing for the possibility of nonlinear dynamics in **v**, the most parsimonious model is linear (shown in Fig. 2). This strongly suggests a connection with the Koopman operator, motivating the present work. The last term *v*_*r*_ is not accurately represented by either linear or polynomial nonlinear models^19^, as is shown in Supplementary Fig. 18.

The structure of the HAVOK model for the Lorenz system is shown in Fig. 2. There is a dominant skew-symmetric structure in the **A** matrix, and the entries are nearly integer valued. In Supplementary Note 4, we demonstrate that the dynamics of a nearby model with exact integer entries qualitatively matches the dynamics of the Lorenz model, including the lobe switching events. This off-diagonal structure and near integrability is the subject of current investigation by colleagues. It was argued in ref. ^35^ that on an example deterministic chaotic system, there is a random dynamical system representation that has the same spectrum and may be used for long-term prediction. The Lorenz system is mixing and does not have a simple spectrum^47^, although it appears that there are functions in the pseudo spectrum that are nearly eigenfunctions of the Koopman operator. Indeed, in the system in ref. ^35^, the Koopman representation has a similar off-diagonal structure to the Lorenz example here.

### HAVOK analysis and prediction in the Lorenz system

**Fig. 3:**
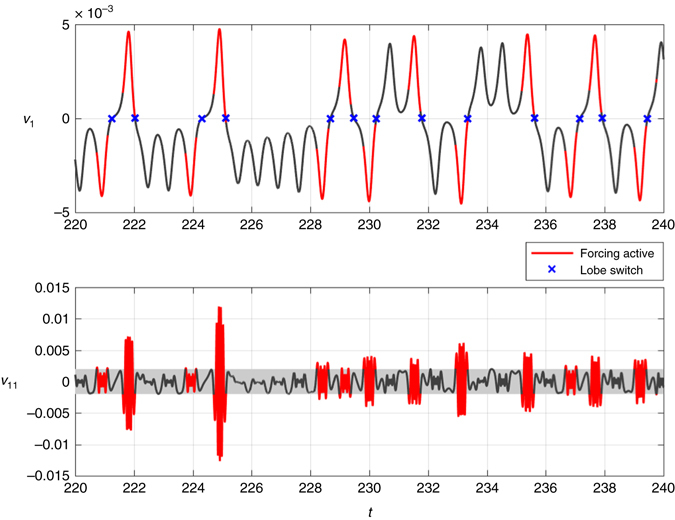
Eigen-time-delay coordinate *v*_1_ of the Lorenz system, colored by the activity of the forcing *v*_*r*_, for *r* = 11. When the forcing is active (*red*), the trajectory is about to switch, and when the forcing is inactive (*gray*), the solution is governed by predominantly linear dynamics corresponding to orbits around one attractor lobe. The forcing is active when $\left| {{v_r}} \right|  >0.002$; this threshold was chosen by trial and error, although it could be varied to sweep out a receiver operating characteristic (ROC) curve to determine the optimal value based on desired sensitivity and specificity

In the case of the Lorenz system, the long tails in the statistics of the forcing signal *v*_*r*_(*t*) correspond to bursting behavior that precedes lobe switching events. It is possible to directly test the power of the forcing signature *v*_*r*_(*t*) to predict lobe switching in the Lorenz system. First, a HAVOK model is trained using data from 200 time units of a trajectory; this results in the basis **U** and the model matrices **A** and **B**. Next, the prediction of lobe switching is tested on a new validation (test) trajectory consisting of the next 1,000 time units (i.e., time *t* = 200 to *t* = 1200). Figure 3 shows 20 time units of this test trajectory. Regions where the forcing term *v*_*r*_ is active are isolated when $|{v_r}|$ is larger than a threshold value; in this case, we choose *r* = 11 and the threshold is 0.002. These regions are colored red in Fig. 3 for *v*_1_ and *v*_*r*_. The remaining portions of the trajectory, when the forcing is small, are colored in dark gray. It is clear by eye that the activity of the forcing precedes lobe switching by nearly one period. During the 1,000 time units of test data there are 605 lobe switching events, of which the HAVOK model correctly identifies 604, for a accuracy of 99.83%. There are likewise 2,047 lobe orbits that do not precede lobe switching, and the HAVOK model identifies 54 false positives at a rate of 2.64%. Note that in this example, both *v*_1_(*t*) and *v*_*r*_(*t*) are computed directly from the time-series using **U**, and are not simulated using the dynamic model. Computing *v*_*r*_ using **U** introduces a short delay of *q*Δ*t* = 0.1 time units; however, forcing activity precedes lobe switching by considerably more than 0.1 time units, so that it is still predictive.

**Fig. 4:**
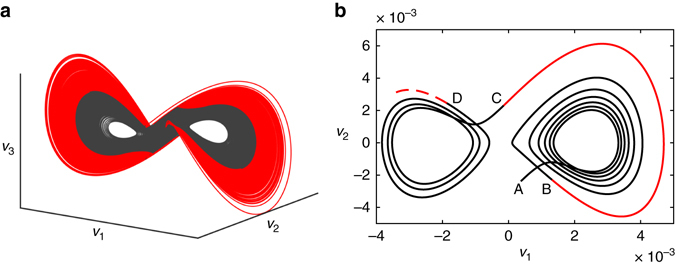
**a** Time-delay embedded attractor of the Lorenz system color-coded by the activity of the forcing term *v*_11_. Trajectories in *gray* correspond to regions where the forcing is small and the dynamics are well approximated by Koopman linear dynamics. The trajectories in *red* indicate that lobe switching is about to occur. **b** Illustration of one intermittent lobe switching event. The trajectory starts at point A, and resides in the basin of the right lobe for six revolutions until B, when the forcing becomes large, indicating an imminent switching event. The trajectory makes one final revolution (*red*) and switches to the *left* lobe C, where it makes three more revolutions. At point D, the activity of the forcing signal *v*_11_ will increase, indicating that switching is imminent

It is important to note that when the forcing term is small, corresponding to the gray portions of the trajectory, the dynamics are largely governed by linear dynamics. Thus, the forcing term in effect distills the essential nonlinearity of the system, indicating when the dynamics are about to switch lobes of the attractor. The same trajectories are plotted in three-dimensions in Fig. 4a, where it can be seen that the nonlinear forcing is active precisely when the trajectory is on the outer portion of the attractor lobes. A single lobe switching event is shown in Fig. 4b, illustrating the geometry of the trajectories.

**Fig. 5:**
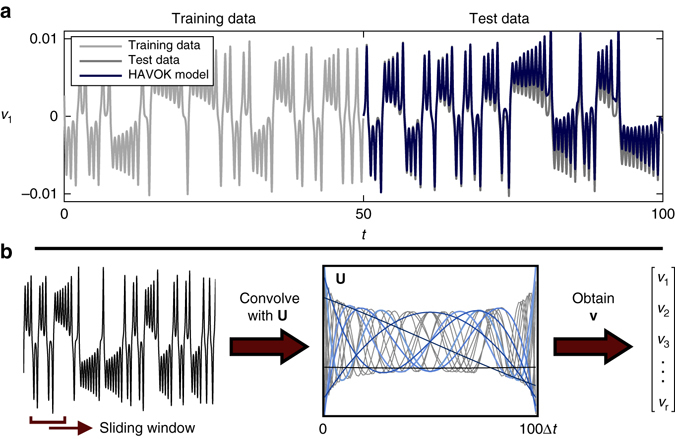
**a** The linear model obtained from training data (*light gray*) may be validated on a new test trajectory. Extracting the *v*_*r*_ signal as an input to the linear model provides an accurate reconstruction (*blue*) of the attractor on the test data (*black*). **b** Illustration of eigen-time-delay modes in **U** for the Lorenz system with *q* = 100 corresponding to a window size of 100 Δ*t* = 0.1 time units. Measurements are convolved with **U** to obtain **v**. The **U** modes resemble polynomials, ordered by energy (i.e., constant, linear, quadratic, etc.). This structure in **U** is common across most of the examples, and provides a criterion to determine the appropriate number of rows *q* in **H** and the rank *r*

Figure 5 shows that the dynamic HAVOK model in (6) generalizes to predict behavior in test data that was not used to train the model. In this figure, a HAVOK model of order *r* = 15 is trained on data from *t* = 0 to *t* = 50, and then simulated on test data from *t* = 50 to *t* = 100. The model captures the main features and lobe transitions, although small errors gradually increase for long times. This model prediction must be run on-line, as it requires access to the forcing signature *v*_*r*_, which may be obtained by multiplying a sliding window of **v**(*t*) with the basis **U**.

### Connection to almost-invariant sets and Perron-Frobenius

The Koopman operator is the dual, or left-adjoint, of the Perron-Frobenius operator, which is also called the transfer operator on the space of probability densities. Thus, Koopman analysis is typically concerned with measurements from a single trajectory, while Perron-Frobenius analysis is concerned with an ensemble of trajectories. Because of the close relationship of the two operators, it is interesting to compare the HAVOK analysis with the almost-invariant sets from the Perron-Frobenius operator. Almost-invariant sets represent dynamically isolated phase space regions, in which the trajectory resides for a long time. These sets are almost invariant under the action of the dynamics and are related to dominant eigenvalues and eigenfunctions of the Perron-Frobenius operator. They can be numerically determined from its finite-rank approximation by discretizing the phase space into small boxes and computing a large, but sparse, transition probability matrix of how initial conditions in the various boxes flow to other boxes in a fixed amount of time; for this analysis, we use the same *q* = 100 for the length of the **U** vectors as in the HAVOK analysis. Following the approach proposed by ref. ^48^, almost-invariant sets can then be estimated by computing the associated reversible transition matrix and level-set thresholding its right eigenvectors.

**Fig. 6:**
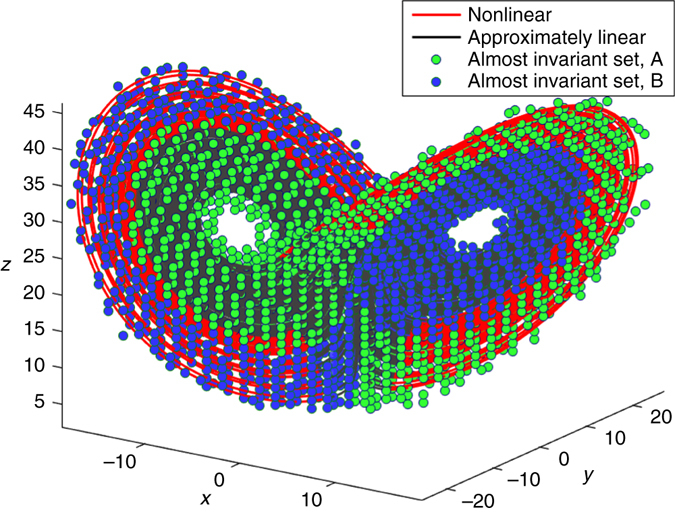
Lorenz attractor visualized using both the HAVOK approximately linear set as well as the Perron-Frobenius almost-invariant sets

The almost-invariant sets of the Perron-Frobenius operator are shown in Fig. 6 for the Lorenz system. There are two sets, each corresponding to the near basin of one attractor lobe as well as the outer basin of the opposing attractor lobe and the bundle of trajectories that connect them. These two almost-invariant sets dovetail to form the complete Lorenz attractor. Underneath the almost-invariant sets, the Lorenz attractor is colored by the thresholded magnitude of the nonlinear forcing term in the HAVOK model, which partitions the attractor into two sets corresponding to regions where the flow is approximately linear (inner black region) and where the flow is strongly nonlinear (outer red region). The boundaries of the almost-invariant sets of the Perron-Frobenius operator closely match the boundaries from the HAVOK analysis.

### Demonstration on examples

**Fig. 7:**
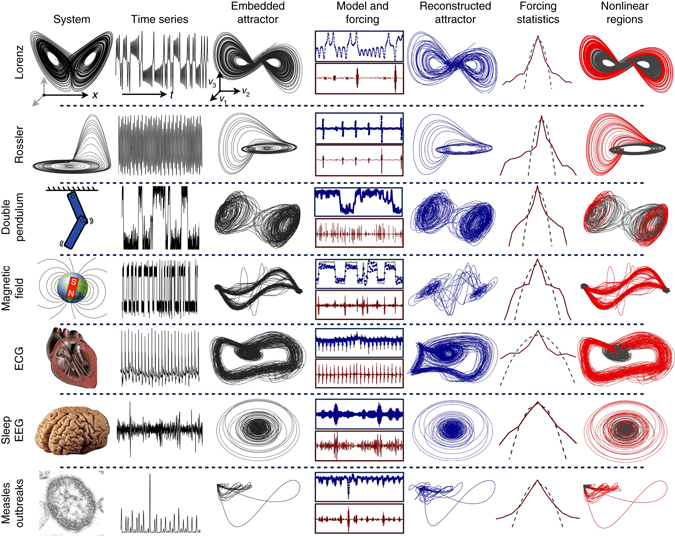
HAVOK analysis applied to a number examples, including analytical systems (Lorenz and Rössler), stochastic magnetic field reversal, and systems characterized from real-world data (electrocardiogram, electroencephalogram, and measles outbreaks). The model is extremely accurate for the first four analytical cases, providing faithful attractor reconstruction and predicting dominant transient and intermittent events. Similarly, in the case of measles outbreaks, the forcing signal is potentially predictive of large transients corresponding to outbreaks. The examples are characterized by nearly symmetric forcing distributions with fat tails (Gaussian forcing is shown in *black dashed line*), corresponding to rare forcing events. Nonlinear measurements *y*(*t*) = *g*(**x**(*t*)) may be used in (4) to enhance features of the embedded attractor. This HAVOK analysis builds on the decades of existing time-delay embedding literature by providing accurate intermittently forced linear regression models for chaotic dynamics. Credit for images in the *left* column: (Earth’s magnetic field) Zureks on Wikimedia Commons; (human heart) Public domain; (human brain) Sanger Brown M.D. on Wikimedia Commons; (measles) CDC/Cynthia S. Goldsmith, William Bellini, Ph.D

The HAVOK analysis is applied to analytic and real-world systems in Fig. 7. More details about each of these systems is presented in Supplementary Note 6, and code for every example is publicly available. The examples span a wide range of systems, including canonical chaotic dynamical systems, such as the Lorenz and Rössler systems, and the double pendulum, which are among the simplest systems that exhibit chaotic motion. As a more realistic example, we consider a stochastically driven simulation of the Earth’s magnetic field reversal^49^, where complex magnetohydrodynamic equations are modeled as a dynamo driven by turbulent fluctuations. In this case, the exact form of the attractor is not captured by the linear model, although the attractor switching, corresponding to magnetic field reversal, is preserved. In the final three examples, we explore the method on data collected from an electrocardiogram (ECG), electroencephalogram (EEG), and recorded measles cases in New York City over a 36 year timespan from 1928 to 1964; sources for all data are provided in Supplementary Note 6.

In each example, the qualitative attractor dynamics are captured, and large transients and intermittent phenomena are highly correlated with the intermittent forcing in the model. These large transients and intermittent events correspond to coherent regions in phase space where the forcing is large (right column of Fig. 7, red). Regions where the forcing is small (black) are well-modeled by a Koopman linear system in delay coordinates. Large forcing often precedes intermittent events (lobe switching for Lorenz system and magnetic field reversal, or bursting measles outbreaks), making this signal strongly correlated and potentially predictive. However, caution must be taken when using time-delay coordinates in streaming or real-time applications, as the HAVOK forcing signature will be delayed by *q*Δ*t*. In the case of the Lorenz system, the HAVOK forcing predicts lobe switching by about 1 time unit, while *q*Δ*t* = 0.1; thus, the prediction still precedes the lobe switching. It is important to note that every model identified and presented here is either neutrally or asymptotically stable. Although we are not aware of theoretical guarantees that data-driven methods like HAVOK will remain stable, it is intuitive that if we sample enough data from a chaotic attractor, the eigenvalues of the models should converge to the unit circle (in discrete-time). In practice, it is certainly possible to obtain unstable models, although this is usually preventable by careful choice of the model order *r*, as discussed above. For example, if the choice of *r* is too large^50^, the model overfits to noise, and is thus prone to instability. In general, sparse regression can have a stabilizing effect by penalizing model terms that are not necessary, preventing overfitting that can lead to instability. In practice, it may also be helpful to add a small amount of numerical diffusion to stabilize models.

## Discussion

In summary, we have presented a data-driven procedure, the HAVOK analysis, to identify an intermittently forced linear system representation of chaos. This procedure is based on machine learning regression, Takens’ embedding, and Koopman theory. In practice, HAVOK first applies DMD or sparse regression (SINDy) to delay coordinates followed by a splitting of variables to handle strong nonlinearities as intermittent forcing; applying DMD to delay coordinates has already been explored in the context of rank-deficient data^42, 43, 51^. The activity of the forcing signal in the Lorenz model is shown to predict lobe switching, and it partitions phase space into coherent linear and nonlinear regions. In the other examples, the forcing signal is correlated with intermittent transient events, such as switching and bursting, and may be predictive.

There are many interesting directions to investigate related to this work. Understanding the skew-symmetric structure of the HAVOK model and the near-integrability of chaotic systems is a topic of ongoing research. Moreover, a detailed mathematical understanding of chaotic systems with continuous spectra will also improve the interpretation of this work. Because the method is data-driven, there are open questions related to the required quantity and quality of data and the resulting model performance. There are also interesting relationships between the number of delays included in the Hankel matrix and the geometry of the resulting embedded attractor. Finally, the use of HAVOK analysis for real-time prediction, estimation, and control is the subject of ongoing work by the authors.

The search for intrinsic or natural measurement coordinates is of central importance in finding simple representations of complex systems, and this will only become increasingly important with growing data. Specifically, intrinsic measurement coordinates can benefit other theoretical and applied work involving Koopman theory^44, 52–56^ and related topics^57–61^. Simple, linear representations of complex systems is a long sought goal, providing the hope for a general theory of nonlinear estimation, prediction, and control. This analysis will hopefully motivate novel strategies to measure, understand, and control^62^ chaotic systems in a variety of scientific and engineering applications.

## Methods

### Choice of model parameters

In practice, there are a number of important considerations when applying HAVOK analysis. Heuristically, there are two main choices that are important in every example: first, choosing the timestep and number of rows, *q*, in the Hankel matrix to obtain a suitable delay embedding basis **U**, and second, choosing the truncation rank *r*, which determines the model order *r*−1. For the first choice, it has been observed that models are more accurate and predictive when the basis **U** resembles polynomials of increasing order, as shown in Fig. 5b or in Supplementary Fig. 11. Decreasing Δ*t* can improve the basis **U** to a point, and then decreasing further has little effect. Similarly, there is a relatively broad range of *q* values that admit a polynomial basis for **U**, and this is chosen in every example. As seen in Supplementary Table 3, for the numerical examples where time is nondimensionalized, the product *q*Δ*t* (i.e., the time window considered in the row direction) is equal to 0.1 time units. For the second choice, there are many important factors to consider when selecting the model order *r*. These factors are explored in detail for the Lorenz system in Supplementary Figs 16 and 17 in Supplementary Note 5, and they are summarized here: model accuracy on both the training data and ideally a hold-out data set not used for training; clear distillation of a forcing signature that is active during important intermittent events and quiescent otherwise; signal to noise in the data; prediction of intermittent events; and desired amount of structure in the resulting linear model. For the Lorenz example, we choose *r* = 15 for Fig. 2, because this is the highest order attainable before numerical roundoff corrupts the model. In this example, higher model order elucidates more structure in the sparse linear model shown in Fig. 2. However, the correlation of the forcing signature with intermittent events is relatively insensitive to model order, and we use a model with order *r* = 11 for prediction in Figs 3 and 4.

### Data availability

All data supporting the findings are available within the article and its Supplementary Information, or are available from the authors upon request. In addition, all code used in this study is available at: http://faculty.washington.edu/sbrunton/HAVOK.zip.

## Electronic supplementary material

Supplementary Information Supplementary Notes, Supplementary Figures, Supplementary Tables and Supplementary References
