# Supplementary material for: Chaos as an intermittently forced linear system
Source: Nat Commun. 2017 May 30;8:19. doi: 10.1038/s41467-017-00030-8 (PMC5449398; doi:10.1038/s41467-017-00030-8)
Supplement: Supplementary file 1 — Supplementary Notes, Supplementary Figures, Supplementary Tables and Supplementary References [file 41467_2017_30_MOESM1_ESM.pdf]

Supplementary Note 1 – Introduction

Nonlinear dynamical systems provide a powerful framework to describe the time evolution of interacting quantities in natural and engineered systems [1]. Dynamical systems have a rich history, spanning centuries of investigation on the leading problems of the day, from predicting the motion of planets [2], to understanding turbulent fluid dynamics [3, 4, 5, 6, 7], to forecasting the changing weather and climate of our own planet [8, 9, 10, 11, 12].

Historically, the two dominant perspectives on dynamical systems have either been geometric or statistical [13]. In the geometric perspective, illustrated in Supplemental Figure 1, the organization and topology of trajectories in phase space provides a qualitative picture of global dynamics and enables detailed quantitative descriptions of local dynamics near fixed points or periodic orbits [1, 14, 15, 16, 17, 18]. Phase space transport is largely mediated by saddle points, and even in relatively simple systems, such as the double pendulum or Lorenz system in Supplemental Figure 1, the dynamics may give rise to chaotic behavior. The statistical perspective trades the analysis of a single trajectory with the description of an ensemble of trajectories, providing a notion of mixing and uncertainty, while balancing the apparent structure and disorder in chaotic systems [19, 20, 21, 22, 23, 24, 25]. Currently, a third operator-theoretic perspective, based on the evolution of measurement functions of the system, is gaining traction. This approach is not new, being introduced in 1931 by Koopman [26], although the recent deluge of measurement data has renewed interest.

Chaotic dynamics describe an important class of systems that have a sensitive dependence on initial conditions, causing nearby trajectories to diverge exponentially in time. This divergent behavior often gives rise to strange attractors that are densely filled by chaotic trajectories. The sensitivity to initial conditions gives chaos a strange flavor of deterministic unpredictability, with increasing uncertainty as a prediction is forecast further into the future. Balancing the intrinsic structure and randomness in chaotic dynamics remains a central challenge, with potential to provide better forecasting and enable effective control strategies in myriad applications. In this work, we leverage the Koopman operator theory to provide a data-driven decomposition of chaotic systems that separates the linear evolution and intermittent forcing.

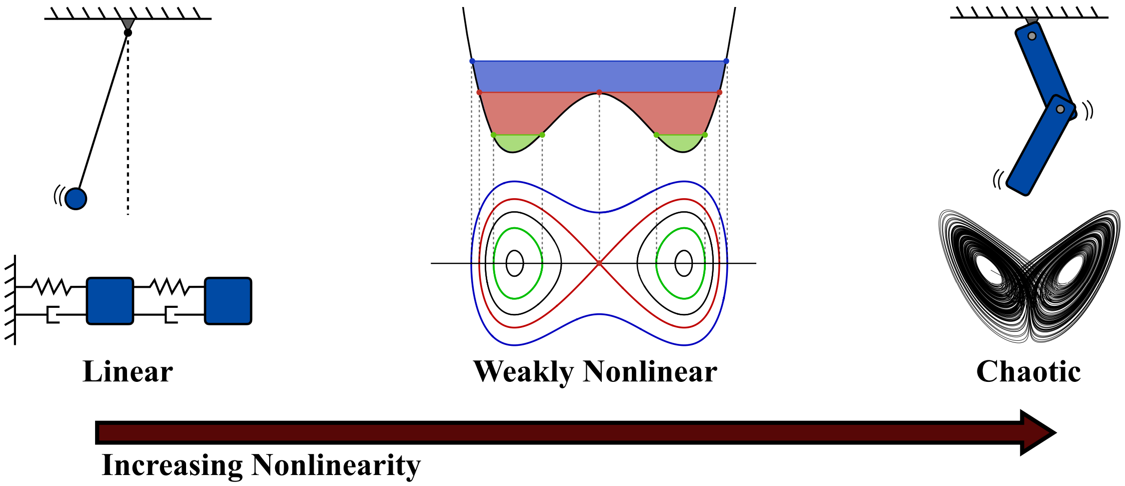

Supplementary Figure 1: Chaotic dynamical systems are often viewed as a progression of increasing nonlinearity.

Supplementary Note 2 – Background

The results in this paper are presented in the context of modern dynamical systems, specifically in terms of the Koopman operator. In this section, we provide a brief overview of relevant concepts in dynamical systems, including a discussion of Koopman operator theory, data-driven dynamical systems regression techniques, and delay embedding theory.

Throughout this work, we will consider dynamical systems of the form:

$$\frac{d}{dt}\mathbf{x}(t) = \mathbf{f}(\mathbf{x}(t)). \tag{1}$$

We will also consider the induced discrete-time dynamical system

$$\mathbf{x}_{k+1} = \mathbf{F}(\mathbf{x}_k) \tag{2}$$

where $\mathbf{x}_k$ may be obtained by sampling the trajectory in (1) discretely in time, so that $\mathbf{x}_k = \mathbf{x}(k\Delta t)$. The discrete-time propagator $\mathbf{F}$ is given by the flow map

$$\mathbf{F}(\mathbf{x}_k) = \mathbf{x}_k + \int_{k\Delta t}^{(k+1)\Delta t} \mathbf{f}(\mathbf{x}(\tau))\, d\tau. \tag{3}$$

The discrete-time perspective is often more natural when considering experimental data.

Koopman operator theory

Koopman spectral analysis was introduced in 1931 by B. O. Koopman [26] to describe the evolution of measurements of Hamiltonian systems, and this theory was generalized in 1932 by Koopman and von Neumann to systems with continuous spectra [27]. Koopman analysis provides an alternative to the more common geometric and statistical perspectives, instead describing the evolution operator that advances the space of measurement functions of the state of the dynamical system. The Koopman operator $\mathcal{K}$ is an infinite-dimensional linear operator that advances measurement functions g of the state $\mathbf{x}$ forward in time according to the dynamics in (2):

$$\mathcal{K}g \triangleq g \circ \mathbf{F} \quad \Longrightarrow \quad \mathcal{K}g(\mathbf{x}_k) = g(\mathbf{x}_{k+1}). \tag{4}$$

Because this is true for all measurement functions g, $\mathcal{K}$ is infinite dimensional and acts on the Hilbert space of functions of the state. For a detailed discussion on the Koopman operator, there are many excellent research articles [28, 29, 30, 31, 32] and reviews [13, 33].

A linear description of nonlinear dynamics is appealing, as many powerful analytic techniques exist to decompose, advance, and control linear systems. However, the Koopman framework trades finite-dimensional nonlinear dynamics for infinite-dimensional linear dynamics. Aside from a few notable exceptions [34, 35], it is rare to obtain analytical representations of the Koopman operator. Obtaining a finite-dimensional approximation (i.e., a matrix $\mathbf{K}$) of the Koopman operator is therefore an important goal of data-driven analysis and control; this relies on a measurement subspace that remains invariant to the Koopman operator [36]. Consider a measurement subspace spanned by measurement functions $\{g_1, g_2, \ldots, g_p\}$ so that for any measurement g in this subspace

$$g = \alpha_1 g_1 + \alpha_2 g_2 + \cdots + \alpha_p g_p \tag{5}$$

then it remains in the subspace after being acted on by the Koopman operator

$$\mathcal{K}g = \beta_1 g_1 + \beta_2 g_2 + \cdots + \beta_p g_p. \tag{6}$$

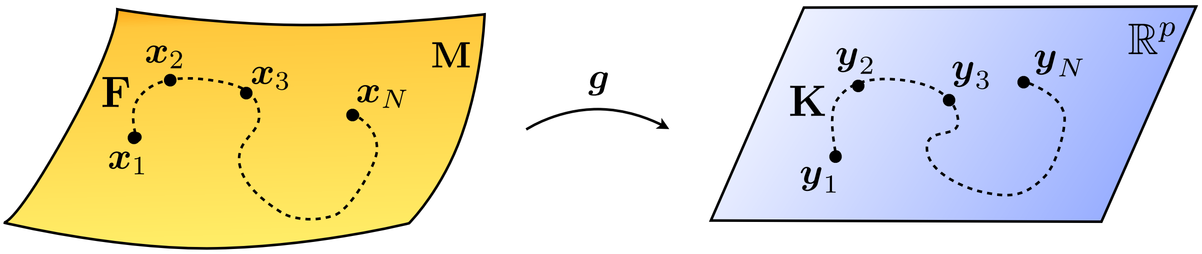

Supplementary Figure 2: Schematic illustrating the ability of the Koopman operator to globally linearize a nonlinear dynamical system with an appropriate choice of observable functions g.

In this case, we may restrict the Koopman operator to this p-dimensional measurement subspace and obtain a $p \times p$ matrix representation, $\mathbf{K}$, as illustrated in Supplemental Figure 2. It has been shown previously that such a representation is useful for prediction and control of certain nonlinear systems that admit finite-dimensional Koopman invariant subspaces [36]. If such a matrix representation exists, it is possible to define a linear system that advances the measurement functions, restricted to the subspace in (5), as follows:

$$\mathbf{y}_{k+1} = \mathbf{K}\mathbf{y}_k, \tag{7}$$

where $\mathbf{y}_k = \begin{bmatrix} g_1(\mathbf{x}_k) & g_2(\mathbf{x}_k) & \cdots & g_p(\mathbf{x}_k) \end{bmatrix}^T$ is a vector of measurements in the invariant subspace evaluated at $\mathbf{x}_k$. Left eigenvectors $\boldsymbol{\xi}$ of $\mathbf{K}$ yield Koopman eigenfunctions according to $\varphi = \boldsymbol{\xi}\mathbf{y}$.

In practice, however, it is extremely challenging to obtain such a representation in terms of a Koopman invariant subspace. Moreover, it is impossible to obtain such an invariant subspace that contains linear measurements of the full state $\mathbf{x}$ for systems with more than one attractor, periodic orbit, and/or fixed point. This is simple to see, since a finite-dimensional linear system does not admit multiple fixed points or attracting structures. In addition, it is not always the case that the Koopman operator even has a discrete spectrum, as in mixing chaotic systems. However, the perspective of a data-driven linear approximation to a dynamical system is still valuable. Linear models can be obtained in entire basins of attraction of fixed points or periodic orbits using Koopman theory with the correct choice of measurement functions [32, 37]. Regression based methods to obtain a finite approximation of the Koopman operator have become standard in the literature, although these methods all rely on a good choice of measurement functions. Identifying good measurement coordinates that approximately or exactly yield linear evolution equations will be one of the central challenges in dynamical systems in the coming years and decades. In the following, we will demonstrate the ability of delay coordinates to provide an approximately invariant measurement space for chaotic dynamics on an attractor.

Data-driven dynamic regression models

With increasingly large volumes of data, it is becoming possible to obtain models using modern regression techniques. This vibrant field will continue to grow as new techniques in machine learning make it possible to extract more information from data. In this section, we provide a brief overview of two leading regression-based system identification techniques: 1) the dynamic mode decomposition (DMD), which provides a best-fit linear operator from high-dimensional snapshot data, and may approximate the Koopman operator in some cases, and 2) the recent sparse identification of nonlinear dynamics (SINDy) algorithm, which produces parsimonious nonlinear models through sparse regression onto a library of nonlinear functions.

Dynamic mode decomposition (DMD)

Dynamic mode decomposition (DMD) was originally introduced in the fluid dynamics community to decompose large experimental or numerical data sets into leading spatiotemporal coherent structures [38]. Shortly after, it was shown that the DMD algorithm provides a practical numerical framework to approximate the Koopman mode decomposition [29]. This connection between DMD and the Koopman operator was further strengthened and justified in a dynamic regression framework [39, 40, 41].

The DMD algorithm seeks a best-fit linear model to relate the following two data matrices

$$\mathbf{X} = \begin{bmatrix} | & | & & | \\ \mathbf{x}_1 & \mathbf{x}_2 & \cdots & \mathbf{x}_{m-1} \\ | & | & & | \end{bmatrix} \qquad \mathbf{X}' = \begin{bmatrix} | & | & & | \\ \mathbf{x}_2 & \mathbf{x}_3 & \cdots & \mathbf{x}_m \\ | & | & & | \end{bmatrix}. \tag{8}$$

The matrix $\mathbf{X}$ contains snapshots of the system state in time, and $\mathbf{X}'$ is a matrix of the same snapshots advanced a single step forward in time. These matrices may be related by a best-fit linear operator $\mathbf{A}$ given by

$$\mathbf{X}' = \mathbf{A}\mathbf{X} \quad \Longrightarrow \quad \mathbf{A} \approx \mathbf{X}'\mathbf{X}^\dagger, \tag{9}$$

where $\mathbf{X}^\dagger$ is the pseudo-inverse, obtained via the singular value decomposition (SVD). The matrix $\mathbf{A}$ is a best-fit linear operator in the sense that it minimizes the Frobenius norm error $\|\mathbf{X}' - \mathbf{A}\mathbf{X}\|_F$.

For systems of moderately large dimension, the operator $\mathbf{A}$ is intractably large, and so instead of obtaining $\mathbf{A}$ directly, we often seek the leading eigendecomposition of $\mathbf{A}$:

1. Take the SVD of $\mathbf{X}$:

$$\mathbf{X} = \mathbf{U}\mathbf{\Sigma}\mathbf{V}^*. \tag{10}$$

   Here, * denotes complex conjugate transpose. Often, only the first r columns of $\mathbf{U}$ and $\mathbf{V}$ are required for a good approximation, $\mathbf{X} \approx \tilde{\mathbf{U}}\tilde{\mathbf{\Sigma}}\tilde{\mathbf{V}}$, where $\tilde{}$ denotes a rank-r truncation.

2. Obtain the $r \times r$ matrix $\tilde{\mathbf{A}}$ by projecting $\mathbf{A}$ onto $\tilde{\mathbf{U}}$:

$$\tilde{\mathbf{A}} = \tilde{\mathbf{U}}^*\mathbf{A}\tilde{\mathbf{U}} = \tilde{\mathbf{U}}^*\mathbf{X}'\tilde{\mathbf{V}}\tilde{\mathbf{\Sigma}}^{-1}. \tag{11}$$

3. Compute the eigendecomposition of $\tilde{\mathbf{A}}$:

$$\tilde{\mathbf{A}}\mathbf{W} = \mathbf{W}\mathbf{\Lambda}. \tag{12}$$

   The eigenvalues in $\mathbf{\Lambda}$ are eigenvalues of the full matrix $\mathbf{A}$.

4. Reconstruct full-dimensional eigenvectors of $\mathbf{A}$, given by the columns of $\mathbf{\Phi}$:

$$\mathbf{\Phi} = \mathbf{X}'\tilde{\mathbf{V}}\tilde{\mathbf{\Sigma}}^{-1}\mathbf{W}. \tag{13}$$

DMD, in its original formulation, is based on linear measurements of the state $\mathbf{x}$ of the system, such as velocity measurements from particle image velocimetry (PIV). This means that the measurement function g is the identity map on the state. Linear measurements are not rich enough for many nonlinear dynamical systems, and so DMD has recently been extended to an augmented measurement vector including nonlinear functions of the state [37]. However, choosing the correct nonlinear measurements that result in an approximately closed Koopman-invariant measurement system is still an open problem. Typically, measurement functions are either determined using information from the dynamical system (i.e., using quadratic nonlinearities for the Navier-Stokes equations), or by a brute-force search in a particular basis of Hilbert space (i.e., searching for polynomial functions or radial basis functions).

Sparse identification of nonlinear dynamics (SINDy)

A recently developed technique, the sparse identification of nonlinear dynamics (SINDy) algorithm, identifies the nonlinear dynamics in (1) from measurement data [42]. The SINDy algorithm uses sparse regression [43] in a nonlinear function space to determine the few active terms in the dynamics. Earlier related methods based on compressed sensing have been used to predict catastrophes in dynamical systems [44]. There are alternative methods that employ symbolic regression (i.e., genetic programming [45]) to identify dynamics [46, 47]. This work is part of a growing literature that is exploring the use of sparsity in dynamics [48, 49, 50] and dynamical systems [51, 52, 53].

The SINDy algorithm is an equation-free method [54] to identify a dynamical system (1) from data, much as in the DMD algorithm above. The basis of the SINDy algorithm is the observation that for many systems of interest, the function $\mathbf{f}$ only has a few active terms, making it sparse in the space of possible functions. Instead of performing a brute-force search for the active terms in the dynamics, sparse regression makes it possible to efficiently identify the few non-zero terms.

To determine the function $\mathbf{f}$ from data, we collect a time-history of the state $\mathbf{x}(t)$ and the derivative $\dot{\mathbf{x}}(t)$; note that $\dot{\mathbf{x}}(t)$ may be approximated numerically from $\mathbf{x}$. The data is sampled at several times $t_1, t_2, \cdots, t_m$ and arranged into two large matrices:

$$\mathbf{X} = \overset{\text{state}\ \longrightarrow}{\begin{bmatrix} x_1(t_1) & x_2(t_1) & \cdots & x_n(t_1) \\ x_1(t_2) & x_2(t_2) & \cdots & x_n(t_2) \\ \vdots & \vdots & \ddots & \vdots \\ x_1(t_m) & x_2(t_m) & \cdots & x_n(t_m) \end{bmatrix}} \Big\downarrow \text{time} \qquad \dot{\mathbf{X}} = \begin{bmatrix} \dot{x}_1(t_1) & \dot{x}_2(t_1) & \cdots & \dot{x}_n(t_1) \\ \dot{x}_1(t_2) & \dot{x}_2(t_2) & \cdots & \dot{x}_n(t_2) \\ \vdots & \vdots & \ddots & \vdots \\ \dot{x}_1(t_m) & \dot{x}_2(t_m) & \cdots & \dot{x}_n(t_m) \end{bmatrix}. \tag{14}$$

Next, we construct an augmented library $\boldsymbol{\Theta}(\mathbf{X})$ consisting of candidate nonlinear functions of the columns of $\mathbf{X}$. For example, $\boldsymbol{\Theta}(\mathbf{X})$ may consist of constant, polynomial and trigonometric terms:

$$\boldsymbol{\Theta}(\mathbf{X}) = \begin{bmatrix} | & | & | & | & & | & | & | & | & \\ \mathbf{1} & \mathbf{X} & \mathbf{X}^{P_2} & \mathbf{X}^{P_3} & \cdots & \sin(\mathbf{X}) & \cos(\mathbf{X}) & \sin(2\mathbf{X}) & \cos(2\mathbf{X}) & \cdots \\ | & | & | & | & & | & | & | & | & \end{bmatrix}. \tag{15}$$

Each column of $\boldsymbol{\Theta}(\mathbf{X})$ is a candidate function for the right hand side of (1). Since only a few of these nonlinearities are likely active in each row of $\mathbf{f}$, sparse regression is used to determine the sparse vectors of coefficients $\boldsymbol{\Xi} = \begin{bmatrix}\boldsymbol{\xi}_1 & \boldsymbol{\xi}_2 & \cdots & \boldsymbol{\xi}_n\end{bmatrix}$ indicating which nonlinearities are active.

$$\dot{\mathbf{X}} = \boldsymbol{\Theta}(\mathbf{X})\boldsymbol{\Xi}. \tag{16}$$

Once $\boldsymbol{\Xi}$ has been determined, a model of each row of the governing equations may be constructed as follows:

$$\dot{\mathbf{x}}_k = \mathbf{f}_k(\mathbf{x}) = \boldsymbol{\Theta}(\mathbf{x}^T)\boldsymbol{\xi}_k. \tag{17}$$

We may solve for $\boldsymbol{\Xi}$ in (16) using sparse regression. In many cases, we may need to normalize the columns of $\boldsymbol{\Theta}(\mathbf{X})$ first to ensure that the restricted isometry property holds [44]; this is especially important when the entries in $\mathbf{X}$ are small, since powers of $\mathbf{X}$ will be minuscule.

Note that in the case the library $\boldsymbol{\Theta}$ contains linear measurements of the state, the SINDy method reduces to a linear regression, closely related to the DMD above, but with transposed notation. The SINDy algorithm also generalizes naturally to discrete-time formulations.

Time-delay embedding

It has long been observed that choosing good measurements is critical to modeling, predicting, and controlling dynamical systems. The concept of observability in a linear dynamical system provides conditions for when the full-state of a system may be estimated from a time-history of measurements of the system, providing a rigorous foundation for dynamic estimation, such as the Kalman filter [55, 56, 57, 58, 59]. Although observability has been extended to nonlinear systems [60], significantly fewer results hold in this more general context.

The Takens embedding theorem [6] provides a rigorous framework for analyzing the information content of measurements of a nonlinear dynamical system. It is possible to enrich a measurement, $y(t)$, with time-shifted copies, $y(t-\tau)$, known as delay coordinates. Under certain conditions, the attractor of a dynamical system in delay coordinates is diffeomorphic to the original attractor. Thus, in some cases it may be possible to reconstruct the entire attractor of a turbulent fluid from a time series of a single point measurement. Similar differential embeddings may be constructed using derivatives of the measurement. Takens embedding theory is related to nonlinear observability [61, 62], providing a connection between these two important fields.

Delay embedding has been widely used to analyze and characterize chaotic systems [63, 64, 65, 66, 67, 68, 69]. Several approaches exist for estimating the embedding dimension for time-delay coordinates based on invariants on the attractor [70], the SVD of the Hankel matrix [71], or using the false neighbors method to evaluate the proximity of points in the reconstructed space [72]. The minimal embedding dimension can generally be reduced by choosing an optimized time delay. The use of generalized delay coordinates are also used for linear system identification with the eigensystem realization algorithm (ERA) [73] and in climate science with the singular spectrum analysis (SSA) [74] and nonlinear Laplacian spectrum analysis (NLSA) [10]. All of these methods are based on a singular value decomposition of a Hankel matrix, which is discussed below.

Hankel matrix analysis

Both the eigensystem realization algorithm (ERA) [73] and the singular spectrum analysis (SSA) [74] are based on the construction of a Hankel matrix from a time series of measurement data. In the following, we will present the theory for a single scalar measurement, although this framework generalizes to multiple input, multiple output (MIMO) problems.

The following Hankel matrix $\mathbf{H}$ is formed from a time series of a measurement $y(t)$:

$$\mathbf{H} = \begin{bmatrix} y(t_1) & y(t_2) & \cdots & y(t_p) \\ y(t_2) & y(t_3) & \cdots & y(t_{p+1}) \\ \vdots & \vdots & \ddots & \vdots \\ y(t_q) & y(t_{q+1}) & \cdots & y(t_m) \end{bmatrix}. \quad (18)$$

Taking the singular value decomposition (SVD) of the Hankel matrix,

$$\mathbf{H} = \mathbf{U}\mathbf{\Sigma}\mathbf{V}^T, \quad (19)$$

yields a hierarchical decomposition of the matrix into eigen time series given by the columns of $\mathbf{U}$ and $\mathbf{V}$. These columns are ordered by their ability to express the variance in the columns and rows of the matrix $\mathbf{H}$, respectively. When the measurement $y(t)$ comes from the impulse response of an observable linear system, then it is possible to use the SVD of the matrix $\mathbf{H}$ to reconstruct an accurate model of the full dynamics. This ERA procedure is widely used in system identification, and it has been recently connected to DMD [40, 75]. In the following, we will generalize system identification using the Hankel matrix to nonlinear dynamical systems via the Koopman analysis.

Supplementary Note 3 – HAVOK analysis on the chaotic Lorenz system

To understand the decomposition of a chaotic system into linear dynamics with intermittent forcing, we illustrate the HAVOK analysis in Supplemental Figure 3 on the chaotic Lorenz system [76]:

$$\dot{x} = \sigma(y - x) \tag{20}$$
$$\dot{y} = x(\rho - z) - y \tag{21}$$
$$\dot{z} = xy - \beta z, \tag{22}$$

with parameters $\sigma = 10$, $\rho = 28$, and $\beta = 8/3$. The Lorenz system is among the simplest and most well-studied examples of a deterministic dynamical system that exhibits chaos. A trajectory of the Lorenz system is shown in Supplemental Figure 4, integrated using the parameters in Supplemental Tables 2 and 3. The trajectory moves along an attractor that is characterized by two lobes, switching back and forth between the two lobes intermittently by passing near a saddle point in the middle of the domain at $(x, y, z) = (0, 0, 0)$.

The panels of Supplemental Figure 3 are provided in more detail below with labels and units. First, a time series of the x variable is measured and plotted in Supplemental Figure 5. By stacking time-shifted copies of this measurement vector as rows of a Hankel matrix, as in (18), it is possible to obtain an eigen-time-delay coordinate system through the singular value decomposition. These eigen-time-delay coordinates, given by the columns of the matrix $\mathbf{V}$, are the most self-similar time series features in the measurement $x(t)$, ordered by the variance they capture in the data.

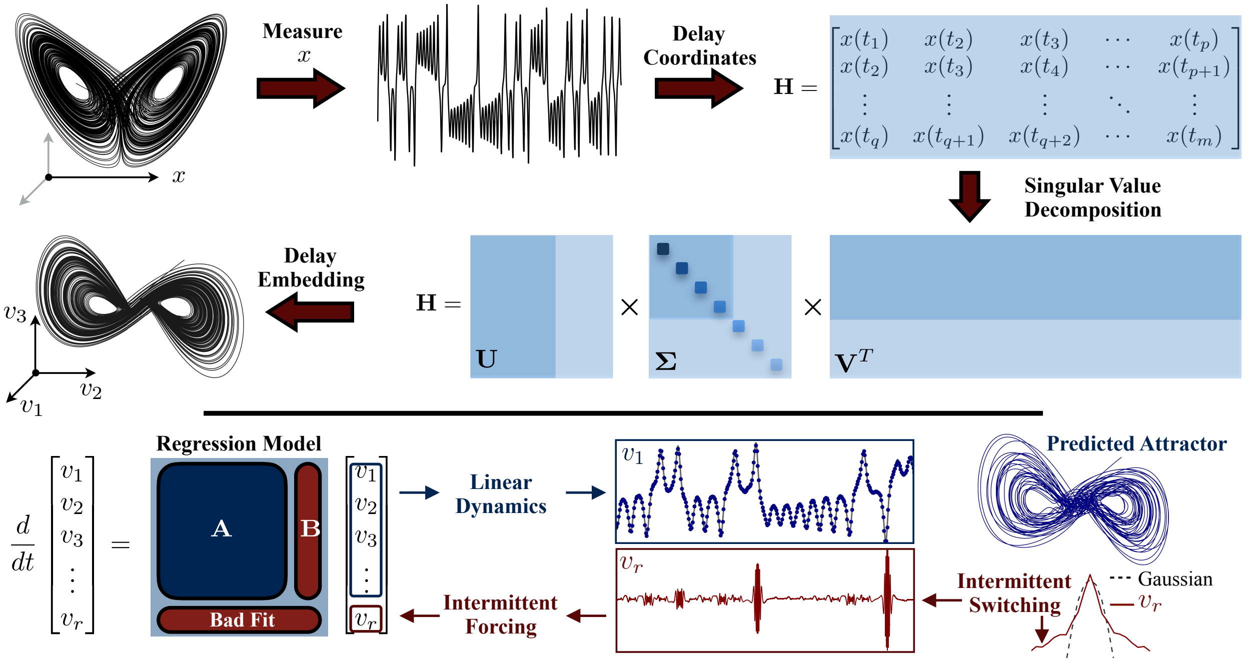

Supplementary Figure 3: Decomposition of chaos into a linear dynamical system with intermittent forcing. First, a time series $x(t)$ of of the chaotic Lorenz system is measured and time-shifted copies are stacked into a Hankel matrix $\mathbf{H}$. Next, the singular value decomposition of $\mathbf{H}$ yields a hierarchical decomposition of eigen time series that characterize the measured dynamics. In these coordinates, given by the columns of $\mathbf{V}$, we obtain a delay-embedded attractor. Finally, a best-fit linear model is obtained on the time-delay coordinates $\mathbf{v}$; the linear fit for the first $r-1$ variables is excellent, but the last coordinate v_r is not well-modeled with linear dynamics. Instead, we consider v_r as an input that forces the first $r-1$ variables. The rare events in the forcing correspond to lobe switching in the chaotic dynamics.

It is known from Takens embedding theory [6] that time-delay coordinates provide an embedding of the scalar measurement $x(t)$ into a higher dimensional space. The resulting attractor in Supplemental Figure 6 is diffeomorphic to the original attractor in Supplemental Figure 4.

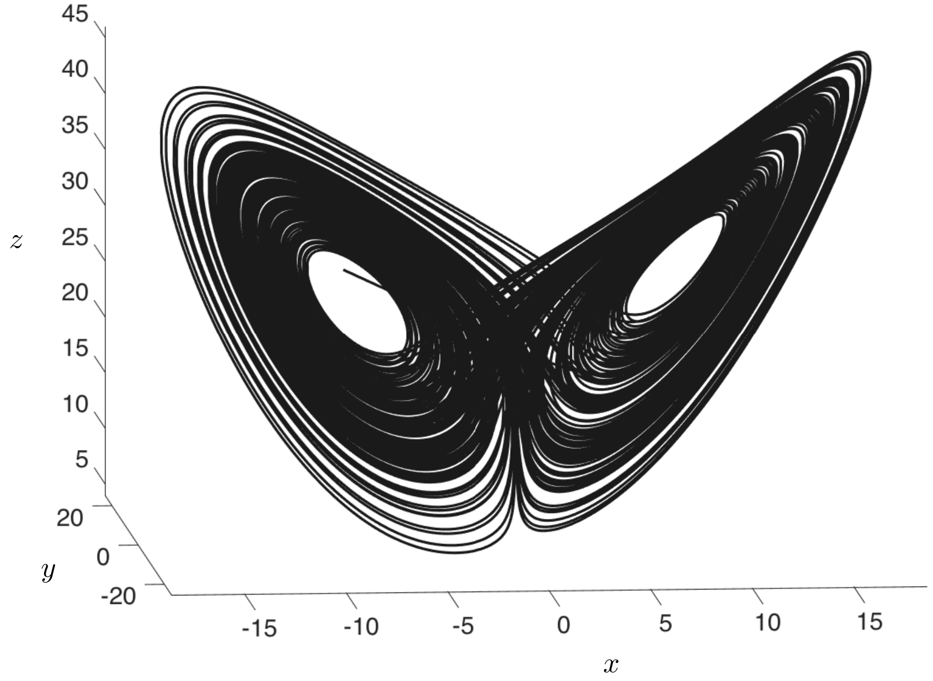

Supplementary Figure 4: Lorenz attractor from (20), simulated using the parameters in Supplemental Tables 2 and 3.

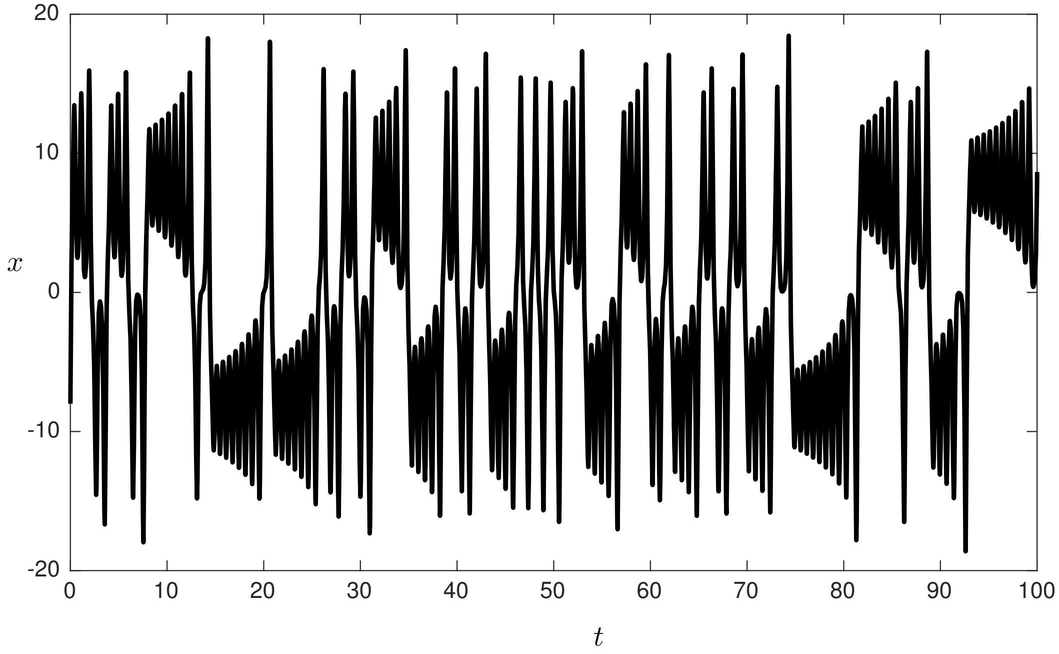

Supplementary Figure 5: A time series of the variable $x(t)$ in the Lorenz system.

Next, a HAVOK model is developed using the time-delay coordinates. In particular, a linear model is obtained for the first 14 coordinates ($v_1, v_2, \ldots, v_{14}$) with linear forcing from the 15th coordinate v_{15}, given by the 15th column of $\mathbf{V}$. This model is depicted schematically in Supplemental Figure 7, and exact values are provided in Supplementary Figure 12. The model may be obtained through a straightforward linear regression procedure, and an additional sparsity penalizing term may be added to eliminate terms in the model with very small coefficients [42]; in the following example, the SINDy algorithm is used with a sparsity knob of $\lambda = 0.025k$ for the v_k equation. The resulting model has a striking skew-symmetric structure, and the terms directly above and below the diagonal are nearly integer multiples of 5. This fascinating structure is explored below.

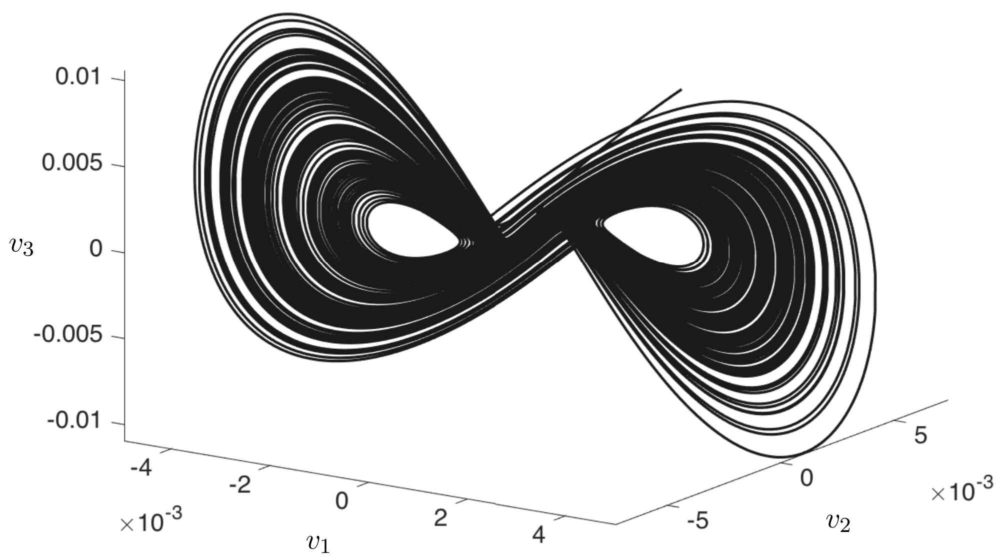

Supplementary Figure 6: Time-delay embedded attractor using the eigen-time-delay coordinates obtained from the singular value decomposition of the Hankel matrix in (18).

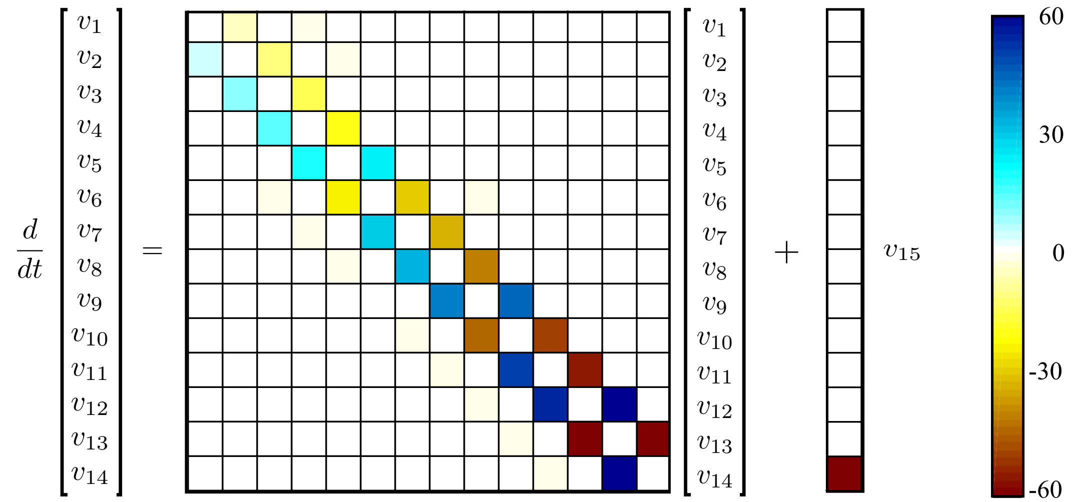

Supplementary Figure 7: HAVOK model obtained on time-delay coordinates of the Lorenz system. Exact coefficients are provided in Supplementary Figure 12.

Using the HAVOK model with the signal v_{15} as an input, it is possible to reconstruct the embedded attractor, as shown in Supplemental Figure 8. Figure 9 shows the model prediction of the dominant time-delay coordinate, v_1, as well as the input forcing signal from v_{15}. From this figure, it is clear that for most times the forcing signal is nearly zero, and this signal begins to burst when the trajectory is about to switch from one lobe of the attractor to another.

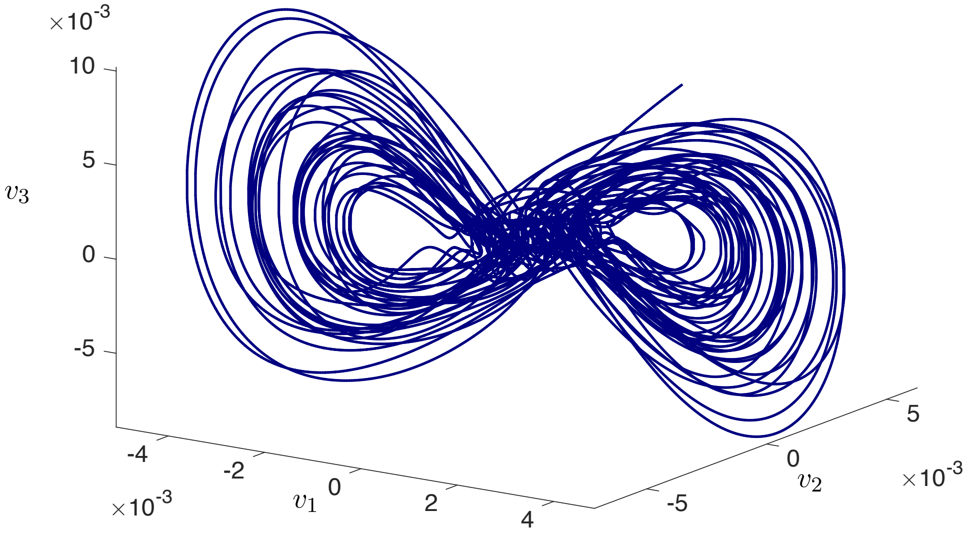

Supplementary Figure 8: Reconstructed embedded attractor using linear HAVOK model with forcing from v_{15}.

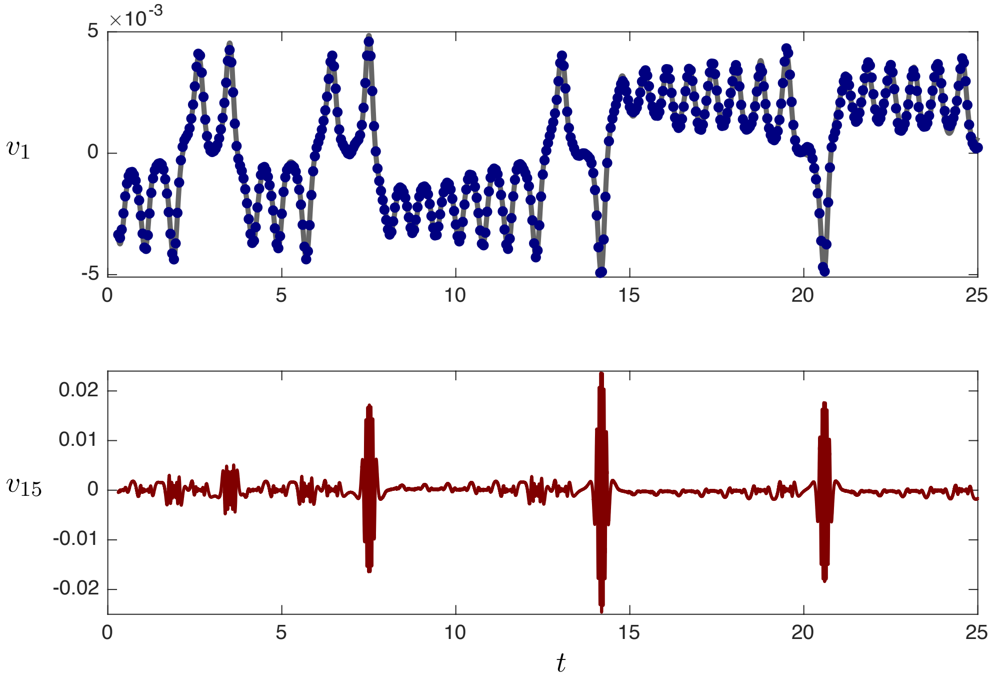

Supplementary Figure 9: Reconstruction of v_1 using linear HAVOK model with forcing from v_{15}.

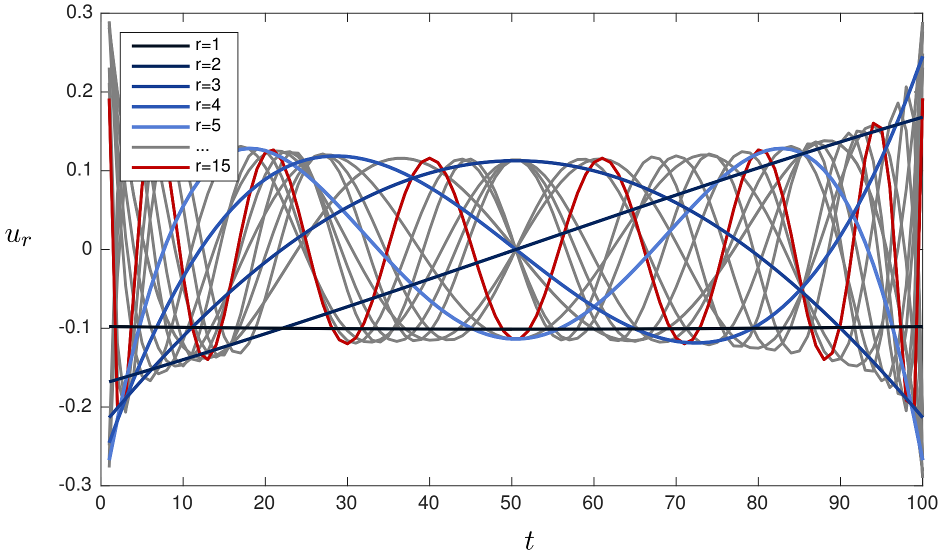

Supplementary Figure 10: Modes u_r (columns of the matrix $\mathbf{U}$), indicating the short-time history that must be convolved with $x(t)$ to obtain v_r.

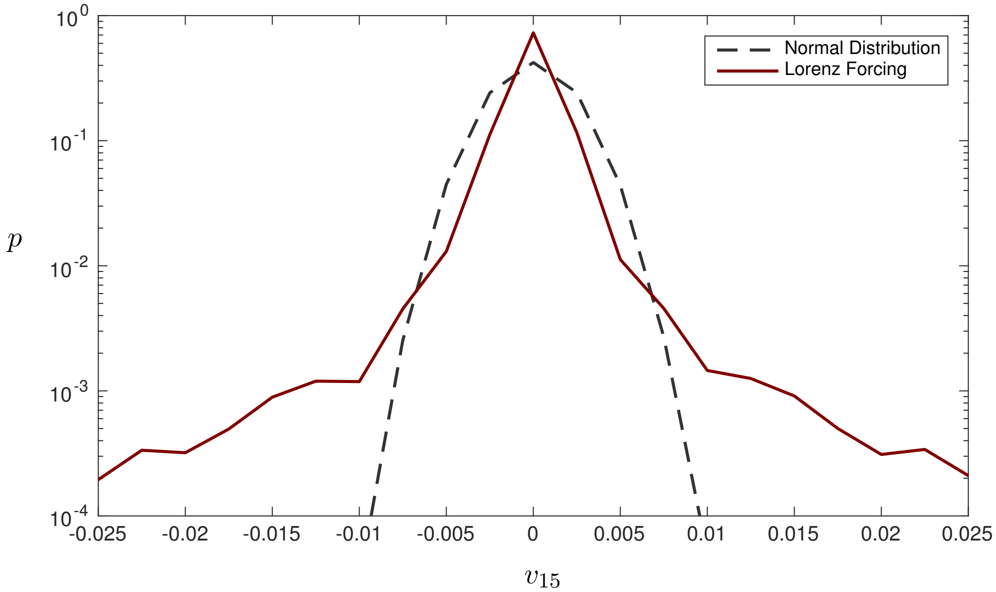

Supplementary Figure 11: Probability density function of the forcing term in v_{15} (red), plotted against the probability density function of an appropriately scaled normal distribution (black dash).

In practice, it is possible to measure v_{15} from a streaming time series of $x(t)$ by convolution with the u_{15} mode (15th column in the matrix $\mathbf{U}$), shown in Supplemental Figure 10. Finally, the probability density function of the forcing term v_{15} is shown in Supplemental Figure 11; the long tails, compared with the normal distribution, indicate the rare intermittent switching events. Dynamic rare events are well-studied in climate and ocean waves [77, 78, 79].

Sparse HAVOK Model of Lorenz System (r=15)

$$
\frac{d}{dt}\begin{bmatrix} v_1 \\ v_2 \\ v_3 \\ v_4 \\ v_5 \\ v_6 \\ v_7 \\ v_8 \\ v_9 \\ v_{10} \\ v_{11} \\ v_{12} \\ v_{13} \\ v_{14} \end{bmatrix} = \begin{bmatrix}
0 & -5.0984 & 0 & -0.1175 & 0 & 0 & 0 & 0 & 0 & 0 & 0 & 0 & 0 & 0 \\
5.1056 & 0 & -9.9391 & 0 & -0.1875 & 0 & 0 & 0 & 0 & 0 & 0 & 0 & 0 & 0 \\
0 & 9.9068 & 0 & -13.5674 & 0 & 0.2450 & 0 & 0 & 0 & 0 & 0 & 0 & 0 & 0 \\
0 & 0 & 13.5045 & 0 & -18.9515 & 0 & 0.3337 & 0 & 0 & 0 & 0 & 0 & 0 & 0 \\
0 & 0 & 0 & 18.8743 & 0 & 23.2686 & 0 & 0.4105 & 0 & 0 & 0 & 0 & 0 & 0 \\
0 & 0 & 0 & 0 & -23.1969 & 0 & -28.9996 & 0 & -0.5105 & 0 & 0 & 0 & 0 & 0 \\
0 & 0 & 0 & 0 & 0 & 28.9710 & 0 & -33.5779 & 0 & 0.5945 & 0 & 0 & 0 & 0 \\
0 & 0 & 0 & 0 & 0 & 0 & 33.5759 & 0 & -39.4090 & 0 & 0.7004 & 0 & 0 & 0 \\
0 & 0 & 0 & 0 & 0 & 0.5710 & 0 & 39.4166 & 0 & 44.0851 & 0 & 0.7875 & 0 & 0 \\
0 & 0 & 0 & 0 & 0 & 0 & -0.6261 & 0 & -44.0173 & 0 & -50.0746 & 0 & 0.8981 & 0 \\
0 & 0 & 0 & 0 & 0 & 0 & 0 & -0.7173 & 0 & 49.9095 & 0 & -54.6529 & 0 & 0.9830 \\
0 & 0 & 0 & 0 & 0 & 0 & 0 & 0 & -0.6723 & 0 & 54.3722 & 0 & 60.8319 & 0 \\
0 & 0 & 0 & 0 & 0 & 0 & 0 & 0 & 0 & -0.7198 & 0 & -60.5289 & 0 & -65.1483 \\
0 & 0 & 0 & 0 & 0 & 0 & 0 & 0 & 0 & 0 & -0.7977 & 0 & 64.9482 & 0
\end{bmatrix}\begin{bmatrix} v_1 \\ v_2 \\ v_3 \\ v_4 \\ v_5 \\ v_6 \\ v_7 \\ v_8 \\ v_9 \\ v_{10} \\ v_{11} \\ v_{12} \\ v_{13} \\ v_{14} \end{bmatrix} + \begin{bmatrix} 0 \\ 0 \\ 0 \\ 0 \\ 0 \\ 0 \\ 0 \\ 0 \\ 0 \\ 0 \\ 0 \\ 1.0964 \\ 0 \\ -71.5168 \end{bmatrix} v_{15}
$$

Neighboring HAVOK Model with Integer Coefficients

$$
\frac{d}{dt}\begin{bmatrix} v_1 \\ v_2 \\ v_3 \\ v_4 \\ v_5 \\ v_6 \\ v_7 \\ v_8 \\ v_9 \\ v_{10} \\ v_{11} \\ v_{12} \\ v_{13} \\ v_{14} \end{bmatrix} = \begin{bmatrix}
0 & -5 & 0 & 0 & 0 & 0 & 0 & 0 & 0 & 0 & 0 & 0 & 0 & 0 \\
5 & 0 & -10 & 0 & 0 & 0 & 0 & 0 & 0 & 0 & 0 & 0 & 0 & 0 \\
0 & 10 & 0 & -15 & 0 & 0 & 0 & 0 & 0 & 0 & 0 & 0 & 0 & 0 \\
0 & 0 & 15 & 0 & -20 & 0 & 0 & 0 & 0 & 0 & 0 & 0 & 0 & 0 \\
0 & 0 & 0 & 20 & 0 & 25 & 0 & 0 & 0 & 0 & 0 & 0 & 0 & 0 \\
0 & 0 & 0 & 0 & -25 & 0 & -30 & 0 & 0 & 0 & 0 & 0 & 0 & 0 \\
0 & 0 & 0 & 0 & 0 & 30 & 0 & -35 & 0 & 0 & 0 & 0 & 0 & 0 \\
0 & 0 & 0 & 0 & 0 & 0 & 35 & 0 & -40 & 0 & 0 & 0 & 0 & 0 \\
0 & 0 & 0 & 0 & 0 & 0 & 0 & 40 & 0 & 45 & 0 & 0 & 0 & 0 \\
0 & 0 & 0 & 0 & 0 & 0 & 0 & 0 & -45 & 0 & -50 & 0 & 0 & 0 \\
0 & 0 & 0 & 0 & 0 & 0 & 0 & 0 & 0 & 50 & 0 & -55 & 0 & 0 \\
0 & 0 & 0 & 0 & 0 & 0 & 0 & 0 & 0 & 0 & 55 & 0 & 60 & 0 \\
0 & 0 & 0 & 0 & 0 & 0 & 0 & 0 & 0 & 0 & 0 & -60 & 0 & -65 \\
0 & 0 & 0 & 0 & 0 & 0 & 0 & 0 & 0 & 0 & 0 & 0 & 65 & 0
\end{bmatrix}\begin{bmatrix} v_1 \\ v_2 \\ v_3 \\ v_4 \\ v_5 \\ v_6 \\ v_7 \\ v_8 \\ v_9 \\ v_{10} \\ v_{11} \\ v_{12} \\ v_{13} \\ v_{14} \end{bmatrix} + \begin{bmatrix} 0 \\ 0 \\ 0 \\ 0 \\ 0 \\ 0 \\ 0 \\ 0 \\ 0 \\ 0 \\ 0 \\ 0 \\ 0 \\ -70 \end{bmatrix} v_{15}
$$

Supplementary Figure 12: HAVOK model for Lorenz system.

Supplementary Note 4 – Structure and integrability of the HAVOK model for the Lorenz system

The HAVOK model for the Lorenz system, provided in Supplementary Figure 12, is highly structured. The **A** matrix of the linear dynamics is nearly skew-symmetric, and the terms directly above and below the diagonal are remarkably close to integer multiples of 5. Intrigued by this structure, we have constructed an approximate system below that represents the idealized structure in the HAVOK model for the Lorenz system:

$$
\frac{d}{dt}\begin{bmatrix} v_1 \\ v_2 \\ v_3 \\ v_4 \\ v_5 \\ v_6 \\ v_7 \\ v_8 \\ v_9 \\ v_{10} \\ v_{11} \\ v_{12} \\ v_{13} \\ v_{14} \end{bmatrix} = \begin{bmatrix}
0 & -5 & 0 & 0 & 0 & 0 & 0 & 0 & 0 & 0 & 0 & 0 & 0 & 0 \\
5 & 0 & -10 & 0 & 0 & 0 & 0 & 0 & 0 & 0 & 0 & 0 & 0 & 0 \\
0 & 10 & 0 & -15 & 0 & 0 & 0 & 0 & 0 & 0 & 0 & 0 & 0 & 0 \\
0 & 0 & 15 & 0 & -20 & 0 & 0 & 0 & 0 & 0 & 0 & 0 & 0 & 0 \\
0 & 0 & 0 & 20 & 0 & 25 & 0 & 0 & 0 & 0 & 0 & 0 & 0 & 0 \\
0 & 0 & 0 & 0 & -25 & 0 & -30 & 0 & 0 & 0 & 0 & 0 & 0 & 0 \\
0 & 0 & 0 & 0 & 0 & 30 & 0 & -35 & 0 & 0 & 0 & 0 & 0 & 0 \\
0 & 0 & 0 & 0 & 0 & 0 & 35 & 0 & -40 & 0 & 0 & 0 & 0 & 0 \\
0 & 0 & 0 & 0 & 0 & 0 & 0 & 40 & 0 & 45 & 0 & 0 & 0 & 0 \\
0 & 0 & 0 & 0 & 0 & 0 & 0 & 0 & -45 & 0 & -50 & 0 & 0 & 0 \\
0 & 0 & 0 & 0 & 0 & 0 & 0 & 0 & 0 & 50 & 0 & -55 & 0 & 0 \\
0 & 0 & 0 & 0 & 0 & 0 & 0 & 0 & 0 & 0 & 55 & 0 & 60 & 0 \\
0 & 0 & 0 & 0 & 0 & 0 & 0 & 0 & 0 & 0 & 0 & -60 & 0 & -65 \\
0 & 0 & 0 & 0 & 0 & 0 & 0 & 0 & 0 & 0 & 0 & 0 & 65 & 0
\end{bmatrix}\begin{bmatrix} v_1 \\ v_2 \\ v_3 \\ v_4 \\ v_5 \\ v_6 \\ v_7 \\ v_8 \\ v_9 \\ v_{10} \\ v_{11} \\ v_{12} \\ v_{13} \\ v_{14} \end{bmatrix} + \begin{bmatrix} 0 \\ 0 \\ 0 \\ 0 \\ 0 \\ 0 \\ 0 \\ 0 \\ 0 \\ 0 \\ 0 \\ 0 \\ 0 \\ -70 \end{bmatrix} v_{15}. \quad (23)
$$

This idealized system is forced with the signal v_{15} from the full Lorenz system, and the dynamic response is shown in Supplemental Figure 13. As shown in the zoom-ins in Figs. 14 and 15, the agreement is remarkably good for the first 50 time units, although the idealized model performance degrades over time, as shown for the final 50 time units.

The eigenvalues of the HAVOK model and the integer-valued approximation are in Supplementary Table 1. There is good agreement between the eigenvalues, with the integer-valued model being exactly integrable, so that trajectories reside on a quasi-periodic orbit. This is to be expected with a Koopman operator model of a dynamical system. A number of the imaginary parts of the eigenvalue pairs are near multiples of each other (e.g., 22.0058 is nearly a multiple of 11.1600). This

is also to be expected in a Koopman model, as integer multiples of an eigenvalue of the Koopman operator are also eigenvalues corresponding to that integer power of the Koopman eigenfunction.

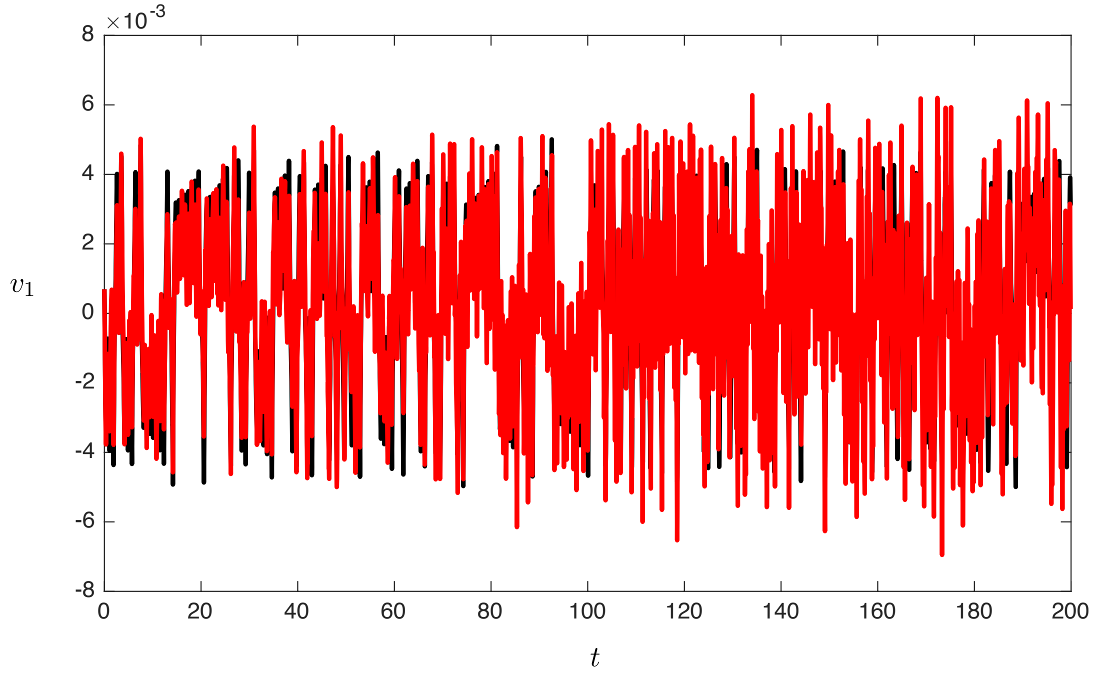

Supplementary Figure 13: Response of idealized HAVOK model with integer coefficients, forced by the [illegible] time series from the Lorenz system.

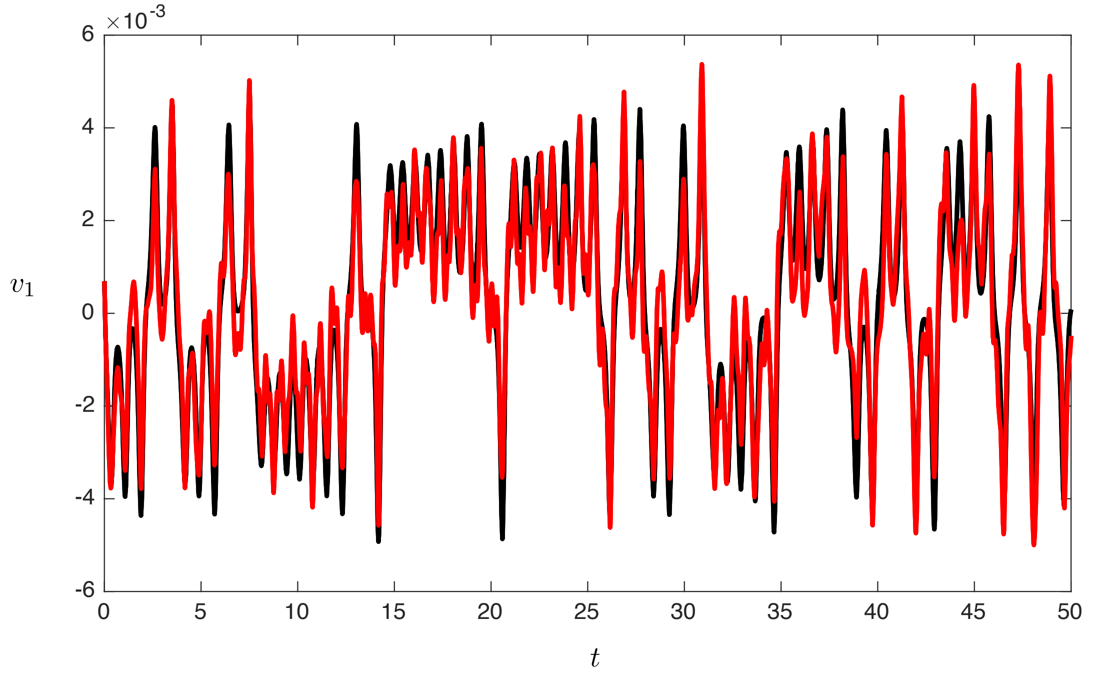

Supplementary Figure 14: First 50 time unites of response of idealized HAVOK model with integer coefficients, forced by the v_{15} time series from the Lorenz system.

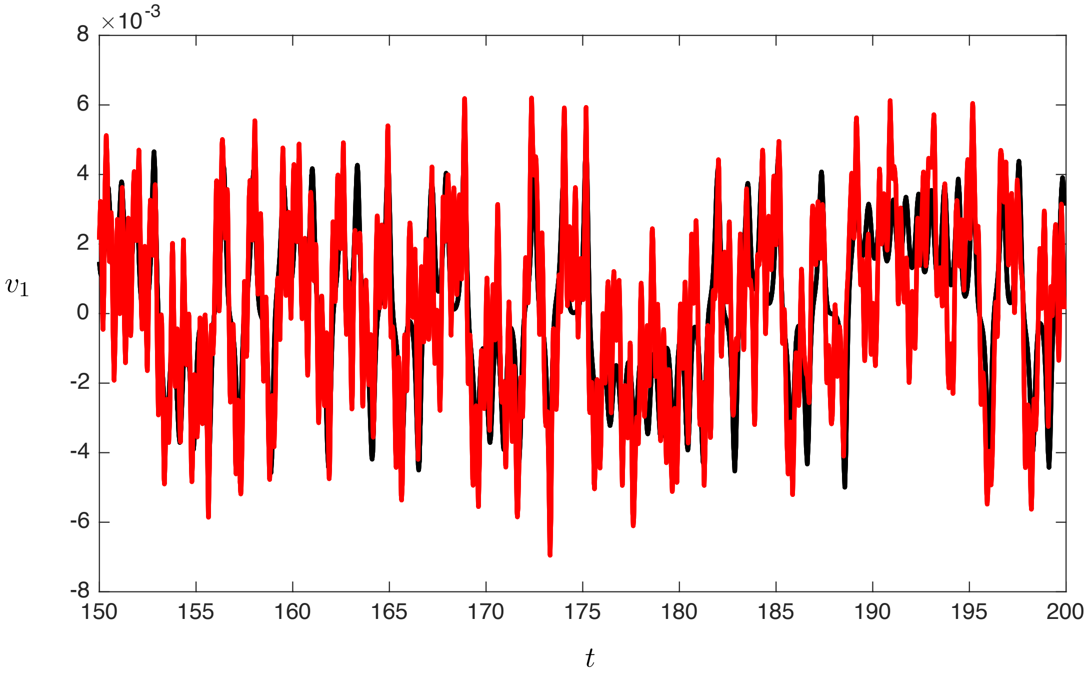

Supplementary Figure 15: Last 50 time unites of response of idealized HAVOK model with integer coefficients, forced by the v_{15} time series from the Lorenz system.

Supplementary Table 1: Eigenvalues of the HAVOK model and integer-valued approximation.

HAVOK Model	Integer-Valued Model
$\pm 2.9703i$	$\pm 3.0725i$
$\pm 11.0788i$	$\pm 11.1600i$
$\pm 21.2670i$	$\pm 22.0058i$
$\pm 34.5458i$	$\pm 35.5714i$
$\pm 51.4077i$	$\pm 52.3497i$
± 72.7790	$\pm 73.5112i$
$\pm 101.6732i$	$\pm 102.2108i$

Supplementary Note 5 – Choice of model order r

There are a number of important factors to consider when choosing the model order, r, including: 1) Model accuracy on training data and on validation data (not used for training); 2) Clear distillation of forcing signature that is active during important events and quiescent otherwise; 3) The signal to noise in the data and singular value decomposition; 4) Prediction of intermittent events and coloring of attractor; and 5.) Structure of the resulting linear model.

The choice of model order r is intimately related to the choice of singular value thresholding of the matrix $\mathbf{H}$. In the Lorenz system, we choose $r = 15$ because it is the highest order model that accurately reconstructs attractor dynamics, retains neutral stability, provides a forcing signature that correlates with important lobe switching events, and has a meaningful sparse model structure. This is purely an artifact of the precision of the data used to train the model. If higher precision data is used, it is possible that a higher order model may be identified. Similarly, if the data is corrupted with noise, then a lower model order must be used. In this case, the optimal singular value hard threshold result of Gavish and Donoho [80] may be used to identify the dominant singular values that are above the noise floor in the data. However, this optimal threshold assumes Gaussian white noise, which may not be realistic for noisy data from a chaotic system; in fact, we find highly non-Gaussian distributions in low-energy singular vectors in many systems, motivating an extension to the Gavish and Donoho result for structured noise distributions.

Fortunately, the main results are not particularly sensitive to the model order. Figure 16 shows the delay embedded attractor colored by forcing activity for a number of different values of r. The qualitative behavior is quite insensitive to the exact model order from about $r = 9$ to $r = 15$, with a slight difference in the attractors for even and odd model order (likely because pure conjugate eigenvalue pairs are only possible for even model order $r - 1$).

For a principled selection of model order, we recommend testing the various models on a hold-

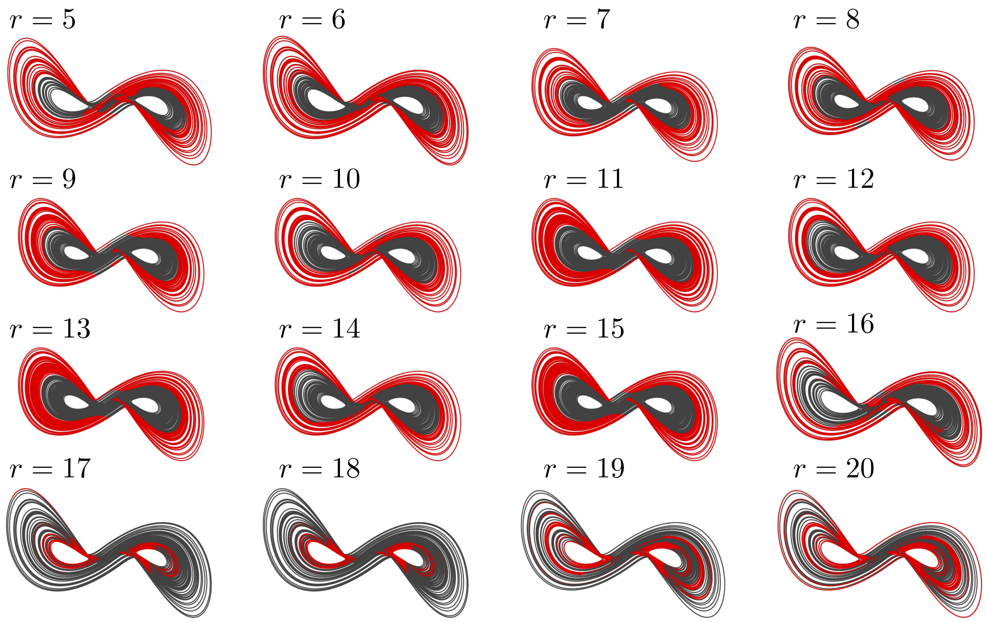

Supplementary Figure 16: Delay-embedded attractor color-coded by the activity of the forcing term v_r for various truncation values r. Note that the odd model orders $r = 9$, $r = 11$, $r = 13$ and $r = 15$ are all quite similar (even model orders from $r = 10$ to $r = 14$ are also similar. However, attractor coloring degrades significantly for $r \geq 17$, along with model accuracy.

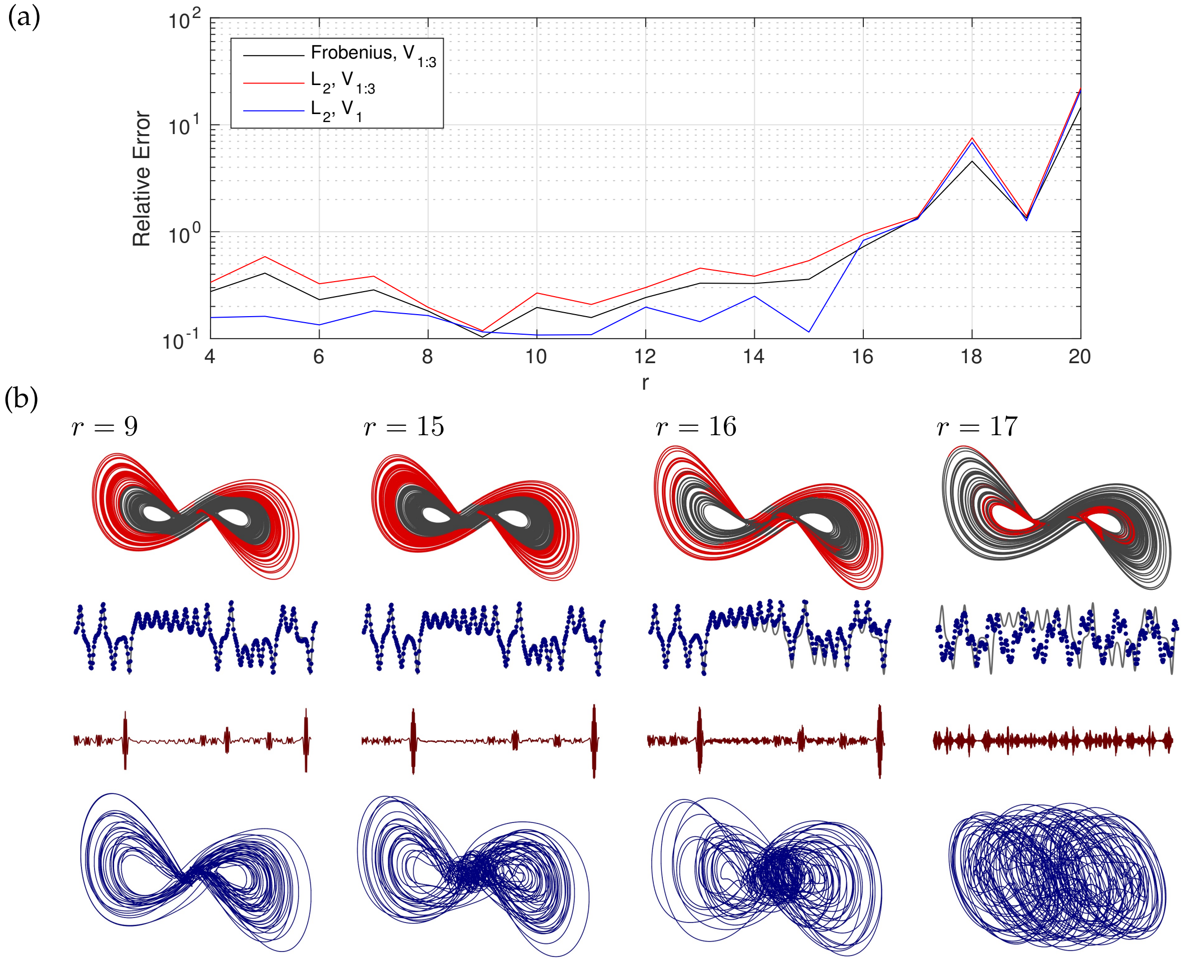

Supplementary Figure 17: (a) The model accuracy on test data (distinct from training data) is plotted against model order r. The reconstruction of the first delay coordinate (V_1) as well as the first three delay coordinates $V_{1:3}$) are shown. (b) The colored attractor, model reconstruction of v_1 given forcing v_r, and the modeled attractor in (v_1, v_2, v_3) are shown for increasing model order r; subplots correspond to Figs. 16, 9, and 8, respectively for $r = 15$, computed on training data.

out data set that was not used for training, as shown in Supplemental Figure 17 (a). In general, it is relatively inexpensive to construct and test models of various orders, as the expensive steps tend to be collecting data and performing the SVD on the Hankel matrix. As the model order is increased, there is little increase in error on the validation data up until $r = 16$, at which point the error climbs significantly; at this point numerical roundoff corrupts the singular vectors. In Supplemental Figure 17 (b), we see that at $r = 17$, the model has large reconstruction errors. Comparing models with $r = 15$ and $r = 16$, we find that the forcing signal becomes noisier in $r = 16$ and is no longer as quiescent between bursts; moreover, the attractor reconstruction (bottom) becomes less well behaved, and the error on the validation set increases. Comparing models with $r = 9$ and $r = 15$, again, the forcing signal with $r = 15$ is slightly more quiescent between lobe switching. In practice, the $r = 9$ model would likely work just as well as the $r = 15$ model, although we choose the higher model order to explore the sparse structure in Supplemental Figure 7.

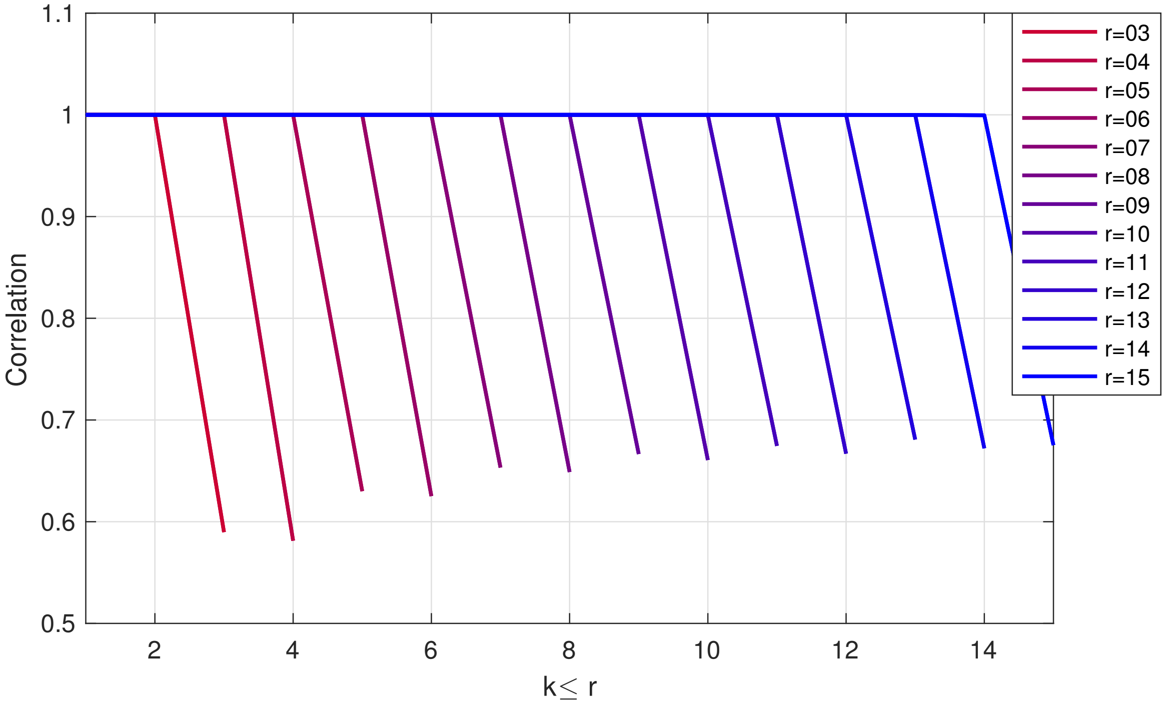

Supplementary Figure 18: Correlation of $\dot{v}_k(t)$ with HAVOK approximation $\mathbf{A}_{k,:}\mathbf{v}(t) + \mathbf{B}v_r(t)$ for each model order r, for the Lorenz system. $\mathbf{A}_{k,:}$ denotes the kth row of $\mathbf{A}$. Note that the model fit is excellent for $k < r$, but the v_r term is never well-modeled with the first r delay coordinates.

Goodness of fit of model with order r

We have stated in the main text that the dynamics of the first $r-1$ eigen-time-delay coordinates are well-modeled by the first r delay coordinates, where the last coordinate $v_r(t)$ is considered a forcing term. We also note that the last delay coordinate $v_r(t)$ is not well-modeled by the first r terms. Supplementary Figure 18 quantifies this goodness of fit of the HAVOK model for the delay coordinates across many model orders, r. For each r, it is seen that the first $r-1$ terms are accurately modeled, and the HAVOK model is highly correlated with the actual derivative data. Similarly, for each model order r, the v_r term is not well-captured by a linear model of the first r terms.

Supplementary Note 6 – Examples

All systems in this paper are described in this section, including details about the equations of motion, data collection, and key characteristics of each system. The simulation parameters for

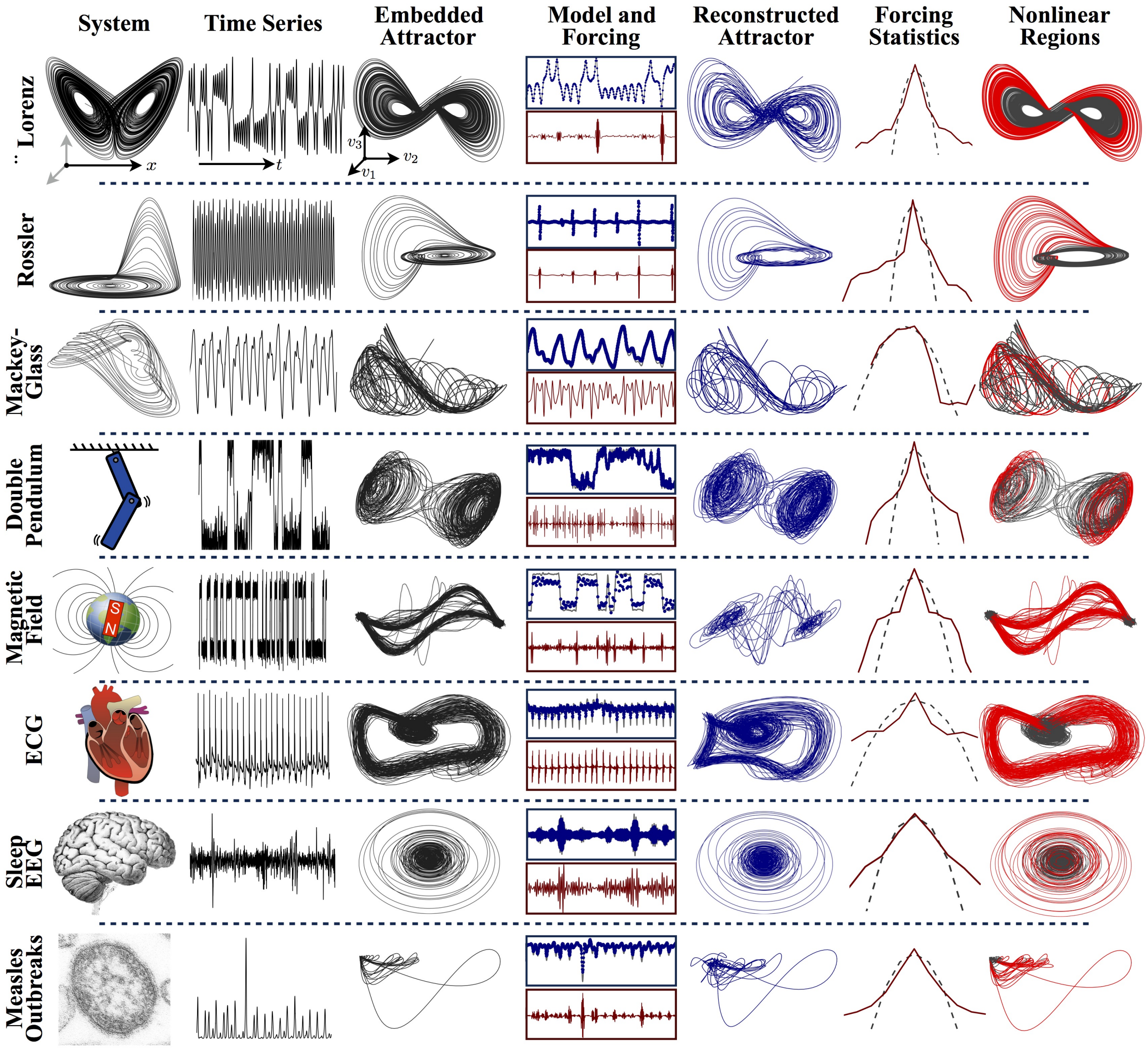

Supplementary Figure 19: Summary of HAVOK analysis applied to numerous examples, including analytical examples (Lorenz and Rössler), delay differential equations (Mackey-Glass), stochastic equations (stochastic magnetic field reversal), and systems characterized from real-world data (electrocardiogram, electroencephalogram, and measles outbreaks). The model prediction is extremely accurate for the first five analytical cases, providing faithful attractor reconstruction and predicting dominant transient and intermittent events. Although the stochastic and data examples are not as accurate, they predict the attractor topology and switching dynamics. In the case of measles outbreaks, the forced linear model predicts large transients corresponding to outbreaks. The majority of the examples have nearly symmetric forcing distributions with fat tails (Gaussian forcing shown in black dashed line), corresponding to rare forcing events. Credit for images in the left column: (Earth's magnetic field) Zureks on Wikimedia Commons; (human heart) Public domain; (human brain) Sanger Brown M.D. on Wikimedia Commons; (measles) CDC/ Cynthia S. Goldsmith, William Bellini, Ph.D.

the numerical examples are in Supplemental Table 2, and the parameters used for the HAVOK analysis are in Supplemental Table 3.

The example systems range from ordinary differential equation (ODE) models to delay differential equations (DDE) and stochastic differential equations (SDE), as well as systems characterized purely by measurement data.

Supplementary Table 2: Parameters used to simulate the various numerical example systems.

System	Parameters	Initial Conditions
**Lorenz**	$\sigma = 10, \rho = 28, \beta = 8/3$	$(-8, 8, 27)$
**Rössler**	$a = 0.1, b = 0.1, c = 14$	$(1, 1, 1)$
**Mackey-Glass**	$\beta = 2, \tau = 2, n = 9.65, \gamma = 1$	0.5
**Double Pendulum**	$l_1 = l_2 = m_1 = m_2 = 1, g = 10$	$(\pi/2, \pi/2, -0.01, -0.005)$
**Magnetic Field**	$B_1 = -0.4605i, B_2 = -1 + 0.12i$, $B_3 = 0.4395i$, $B_4 = -0.06 - 0.12i$, $b_1 = b_4 = 0.25$, and $b_2 = b_3 = 0.07$, $\mu = \nu = 0$	0.1

Supplementary Table 3: Summary of systems and HAVOK analysis parameters for each example. *For the measles data, the original 431 data samples are spaced 1 month apart; this data is interpolated at 0.1 month resolution, and the new data is stacked into $q = 50$ rows.

System	Type	Measured Variable	# Samples m	Time step Δt	# Rows in H q	Rank r	Energy in r modes (%)
**Lorenz**	ODE	$x(t)$	200,000	0.001	100	15	100
**Rössler**	ODE	$x(t)$	500,000	0.001	100	6	99.9999997
**Mackey-Glass**	DDE	$x(t)$	100,000	0.001	100	4	99.9999
**Double Pendulum**	ODE	$\sin(\theta_1(t))$	250,000	0.001	100	5	99.997
**Magnetic Field**	SDE	$\text{Re}(A)$	100,000	1 year	100	4	55.2
**ECG**	Data	Voltage	45,000	0.004 s	25	5	67.4
**Sleep EEG**	Data	Voltage	100,000	0.01 s	1,000	4	7.7
**Measles Outbreak**	Data	Cases	431	1 month	50*	9	99.78

Chaotic Lorenz system

The Lorenz system [76] is a canonical model for chaotic dynamics:

$$\dot{x} = \sigma(y - x) \tag{24}$$
$$\dot{y} = x(\rho - z) - y \tag{25}$$
$$\dot{z} = xy - \beta z. \tag{26}$$

Rössler system

The Rössler system is given by

$$\dot{x} = -y - z \tag{27}$$
$$\dot{y} = x + ay \tag{28}$$
$$\dot{z} = b + z(x - c) \tag{29}$$

This system exhibits interesting dynamics, whereby the trajectory is characterized by oscillation in the $x-y$ plane punctuated by occasional transient growth and decay in the z direction. We refer

to this behavior as bursting, as it corresponds to a transient from an attractor to itself, as opposed to the lobe switching between attractor lobes in the Lorenz system.

Mackey-Glass delay differential equation

The Mackey-Glass equation is a canonical example of a delay differential equation, given by

$$\dot{x}(t) = \beta \frac{x(t-\tau)}{1 + x(t-\tau)^n} - \gamma x(t), \tag{30}$$

with $\beta = 2$, $\tau = 2$, $n = 9.65$, and $\gamma = 1$. The current time dynamics depend on the state $x(t-\tau)$ at a previous time τ in the past.

Double pendulum

The double pendulum is among the simplest physical systems that exhibits chaos. The numerical simulation of the double pendulum is sensitive, and we implement a variational integrator based on the Euler-Lagrange equations

$$\frac{d}{dt}\frac{\partial L}{\partial \dot{\mathbf{q}}} - \frac{\partial L}{\partial \mathbf{q}} = 0 \tag{31}$$

where the Lagrangian $L = T - V$ is the kinetic (T) minus potential (V) energy. For the double pendulum, $\mathbf{q} = \begin{bmatrix}\theta_1 & \theta_2\end{bmatrix}^T$, and the Lagrangian becomes:

$$L = T - V = \frac{1}{2}(m_1 + m_2) l_1 \dot{\theta}_1^2 + \frac{1}{2} m_2 l_2^2 \dot{\theta}_2^2 + m_2 l_1 l_2 \dot{\theta}_1 \dot{\theta}_2 \cos(\theta_1 - \theta_2) \tag{32}$$

$$- (m_1 + m_2) l_1 g (1 - \cos(\theta_1)) - m_2 l_2 g \left(1 - \cos(\theta_2)\right). \tag{33}$$

We integrate the equations of motion with a variational integrator derived using a trapezoidal approximation to the action integral:

$$\delta \int_a^b L(\mathbf{q}, \dot{\mathbf{q}}, t) dt = 0. \tag{34}$$

Because the mean of θ_1 drifts after a revolution, we use $x(t) = \cos(2\theta_1(t))$ as a measurement.

Earth's magnetic field reversal

The Earth's magnetic field is known to reverse over geological time scales [81, 82]. Understanding and predicting these rare events is an important challenge in modern geophysics. It is possible to model the underlying dynamics that give rise to magnetic field switching by considering the turbulent magnetohydrodynamics inside the Earth. A simplified model [83, 84, 85] may be obtained by modeling the turbulent fluctuations as stochastic forcing on a dynamo.

This is modeled by the following differential equation in terms of the magnetic field A:

$$\frac{d}{dt} A = \mu A + \nu \bar{A} + B_1 A^3 + B_2 A^2 \bar{A} + B_3 A \bar{A}^2 + B_4 \bar{A}^3 + f \tag{35}$$

where $\bar{A}$ is the complex conjugate of A and the stochastic forcing f is given by

$$f = (b_1 \xi_1 + i b_3 \xi_3) \operatorname{Re}(A) + (b_2 \xi_2 + i b_4 \xi_4) \operatorname{Im}(A). \tag{36}$$

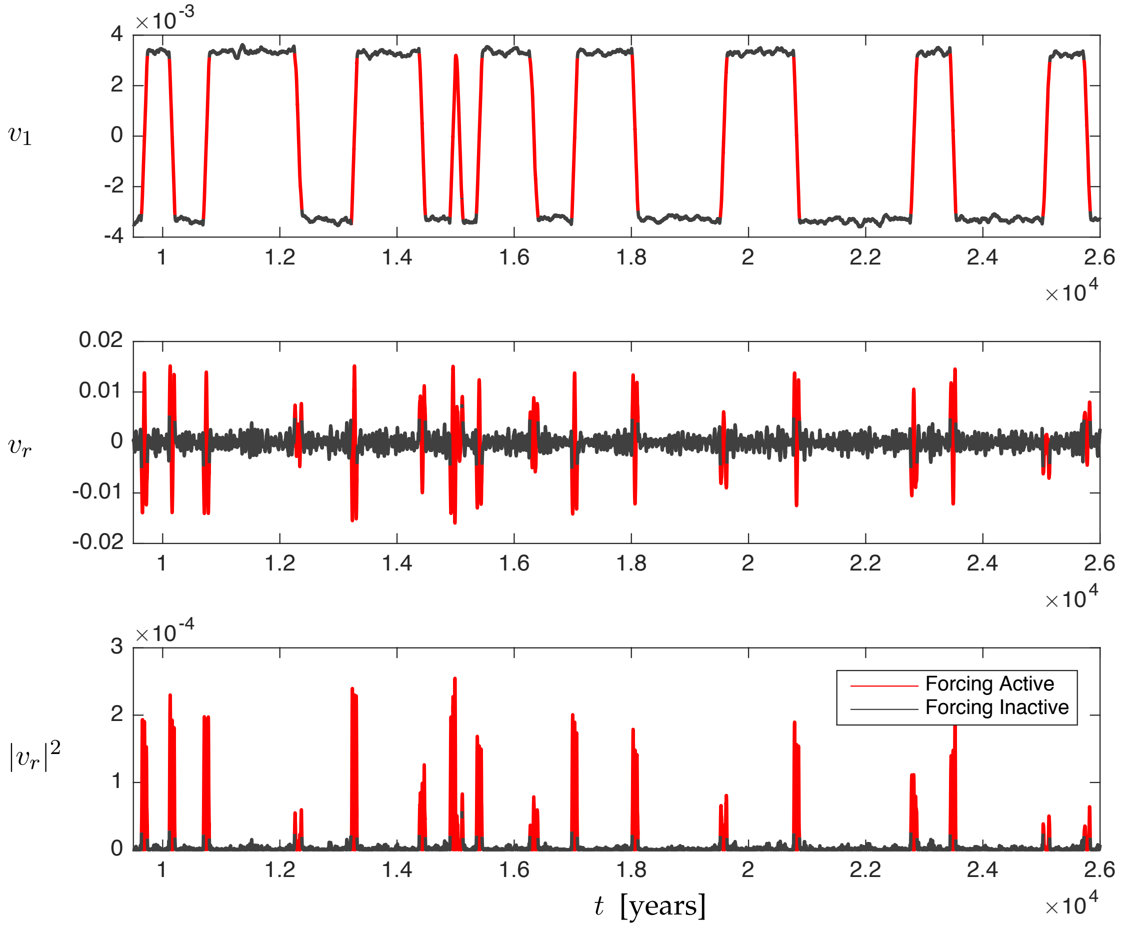

Supplementary Figure 20: Prediction of Earth's magnetic field reversal using the forcing term v_r. Zoom-in of field reversal events in Supplemental Figure 21.

The variables ξ are Gaussian random variables with a standard deviation of 5. The other parameters are given by $\mu = 1$, $\nu = 0$, $B_1 = -0.4605i$, $B_2 = -1 + 0.12i$, $B_3 = 0.4395i$, $B_4 = -0.06 - 0.12i$, $b_1 = b_4 = 0.25$, and $b_2 = b_3 = 0.07$.

Figure 20 shows the v_1 and v_r eigen time series for the Earth magnetic field reversal data. The signal in v_1 accurately represents the global field switching dynamics. Color-coding the trajectory by the magnitude of the forcing in v_r, it is clear that the field reversal events correspond to large forcing. Analyzing two of these field switching events more closely in Supplemental Figure 21, it appears that the forcing signal becomes active before the signal in v_1 exceeds the expected variance. This means that the forcing signal provides a prediction of field reversal before there is a clear statistical signature in the dominant v_1 time series.

Electrocardiogram (ECG)

An electrocardiogram (ECG) measures the electrical activity of the heart, producing the characteristic spiking pulses associated with each heartbeat. Data from ECG has long been analyzed using delay embeddings to quantify the fractal dimension of ECG recordings, among other quantities [86, 87]. In this analysis, we use the ECG signal `qtdb/sel102` that was used in [88], adapted

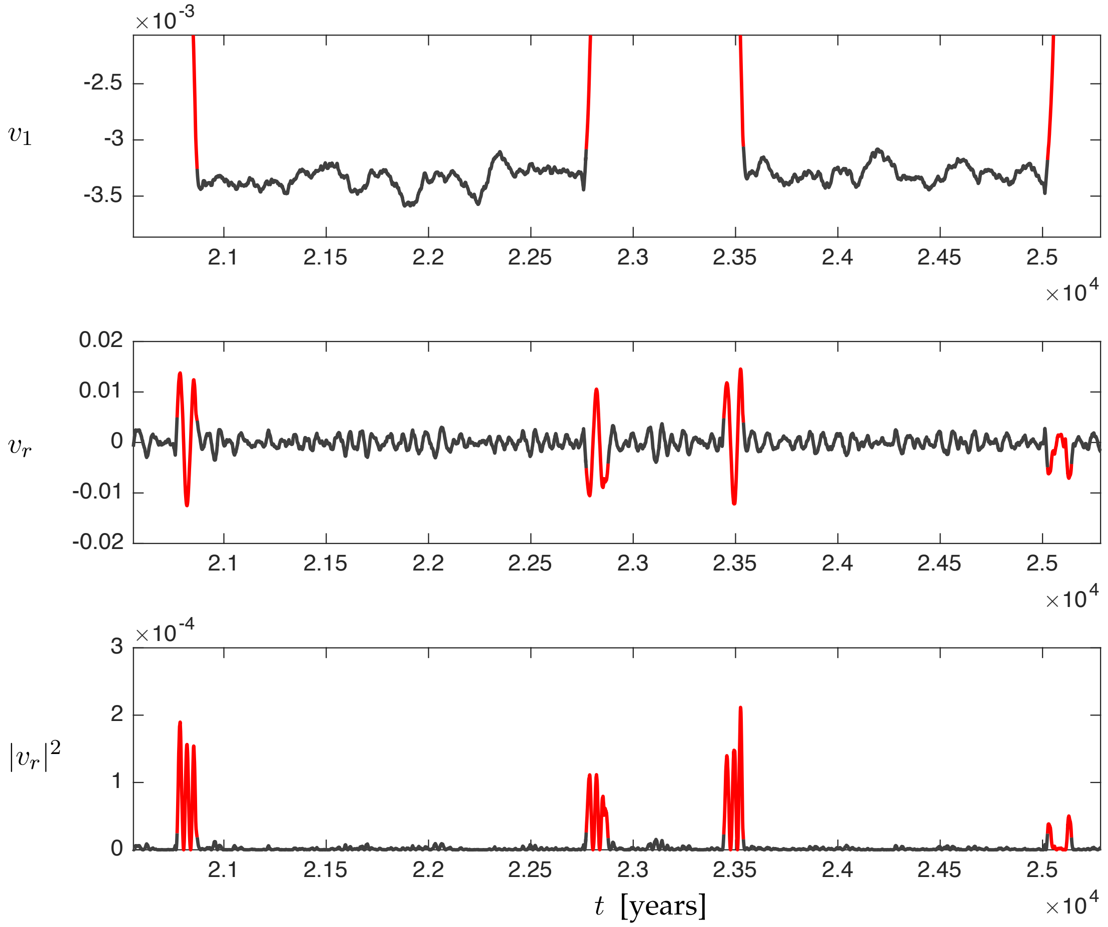

Supplementary Figure 21: Prediction of Earth's magnetic field reversal using the forcing term v_r.

from the PhysioNet database [89, 90]. This signal corresponds to $T = 380$ seconds of data with a sampling rate of 250 Hz.

Electroencephalogram (EEG)

An electroencephalogram (EEG) is a nonintrusive measurement of brain activity achieved through electrodes placed on the scalp. A time series of voltage is recorded from these electrodes, and although typically the spectral content of EEG is analyzed, there are also numerous examples of time series EEG analysis [91, 92, 93, 94, 95]. Although it is possible to obtain vast quantities of data, possibly using an array of electrodes, the voltage signal is only a rough proxy for brain activity, as signals must pass through thick layers of dura, cerebrospinal fluid, skull, and scalp.

Data is available at [90, 96]: https://physionet.org/pn4/sleep-edfx/

Measles outbreaks

Time series analysis has long been applied to understand and model disease epidemics. Measles outbreaks have been particularly well studied, partially because of the wealth of accurate historical data. The seminal work of Sugihara and May [65] use Takens embedding theory to analyze Measles outbreaks. It was also shown that Measles outbreaks are chaotic [97]. Here, we use data for Measles outbreaks in New York City (NYC) from 1928 to 1964, binned every 2 weeks [98].

Figure 22 shows the v_1 and v_r eigen time series for the Measles outbreaks data. The v_1 signal provides a signature for the severity of the outbreak, with larger negative values corresponding to more cases of Measles. The forcing signal accurately captures many of the outbreaks, most notably the largest outbreak after 1940. This outbreak was preceded by two small dips in v_1, which may have resulted in false positives using other prediction methods. However, the forcing signal becomes large directly preceding the outbreak.

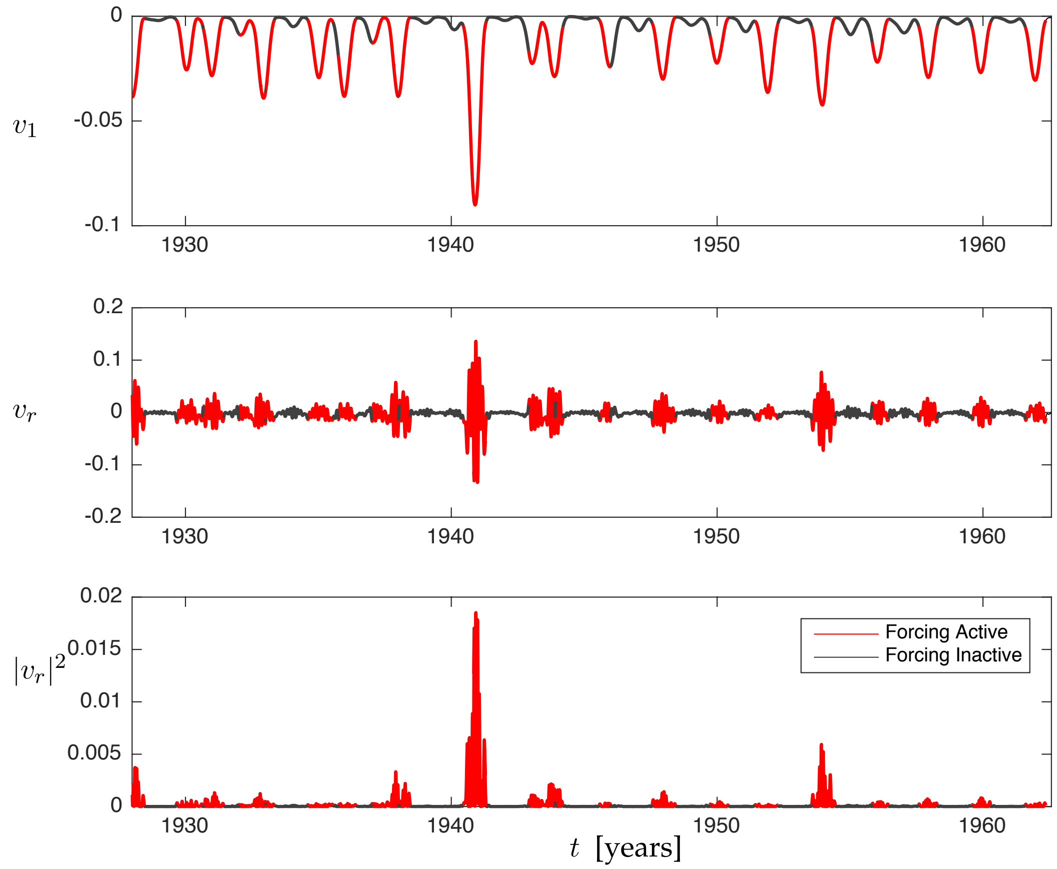

Supplementary Figure 22: Prediction of Measles outbreaks using the forcing term v_r.

Supplementary References

[1] Guckenheimer, J. & Holmes, P. *Nonlinear oscillations, dynamical systems, and bifurcations of vector fields*, vol. 42 of *Applied Mathematical Sciences* (Springer, 1983).

[2] Poincaré, H. Sur le probleme des trois corps et les équations de la dynamique. *Acta Mathematica* **13**, A3–A270 (1890).

[3] Kolmogorov, A. On degeneration (decay) of isotropic turbulence. *Dokl. Akad. Nauk SSSR* **31**, 538–540 (1941).

[4] Kolmogorov, A. Dissipation of energy in locally isotropic turbulence. *Dokl. Akad. Nauk SSSR* **32**, 16–18 (1941). (translated and reprinted 1991 in Proceedings of the Royal Society A **434**, 15–17).

[5] Kolmogorov, A. The local structure of turbulence in incompressible viscous fluid for very large Reynolds number. *Dokl. Akad. Nauk SSSR* **30**, 9–13 (1941). (translated and reprinted 1991 in Proceedings of the Royal Society A **434**, 9–13).

[6] Takens, F. Detecting strange attractors in turbulence. *Lecture Notes in Mathematics* **898**, 366–381 (1981).

[7] Brunton, S. L. & Noack, B. R. Closed-loop turbulence control: Progress and challenges. *Applied Mechanics Reviews* **67**, 050801–1–050801–48 (2015).

[8] Majda, A. J. & Harlim, J. Information flow between subspaces of complex dynamical systems. *PNAS* **104**, 9558–9563 (2007).

[9] Majda, A. J. & Harlim, J. Physics constrained nonlinear regression models for time series. *Nonlinearity* **26**, 201 (2012).

[10] Giannakis, D. & Majda, A. J. Nonlinear Laplacian spectral analysis for time series with intermittency and low-frequency variability. *PNAS* **109**, 2222–2227 (2012).

[11] Sapsis, T. P. & Majda, A. J. Statistically accurate low-order models for uncertainty quantification in turbulent dynamical systems. *PNAS* **110**, 13705–13710 (2013).

[12] Majda, A. J. & Lee, Y. Conceptual dynamical models for turbulence. *PNAS* **111**, 6548–6553 (2014).

[13] Budišić, M., Mohr, R. & Mezić, I. Applied Koopmanism a). *Chaos: An Interdisciplinary Journal of Nonlinear Science* **22**, 047510 (2012).

[14] Marsden, J. E. & McCracken, M. *The Hopf bifurcation and its applications*, vol. 19 (Springer-Verlag, 1976).

[15] Abraham, R., Marsden, J. E. & Ratiu, T. *Manifolds, Tensor Analysis, and Applications*, vol. 75 of *Applied Mathematical Sciences* (Springer-Verlag, 1988).

[16] Chorin, A. J. & Marsden, J. E. *A mathematical introduction to fluid mechanics*, vol. 3 (Springer, 1990).

[17] Marsden, J. E. & Ratiu, T. S. *Introduction to mechanics and symmetry* (Springer-Verlag, 1999), 2nd edn.

[18] Koon, W. S., Lo, M. W., Marsden, J. E. & Ross, S. D. Heteroclinic connections between periodic orbits and resonance transitions in celestial mechanics. *Chaos: An Interdisciplinary Journal of Nonlinear Science* **10**, 427–469 (2000).

[19] Dellnitz, M., Froyland, G. & Junge, O. The algorithms behind GAIO—set oriented numerical methods for dynamical systems. In *Ergodic theory, analysis, and efficient simulation of dynamical systems*, 145–174 (Springer, 2001).

[20] Dellnitz, M. & Junge, O. Set oriented numerical methods for dynamical systems. *Handbook of dynamical systems* **2**, 221–264 (2002).

[21] Dellnitz, M. *et al.* Transport in dynamical astronomy and multibody problems. *International Journal of Bifurcation and Chaos* **15**, 699–727 (2005).

[22] Froyland, G. & Padberg, K. Almost-invariant sets and invariant manifolds – connecting probabilistic and geometric descriptions of coherent structures in flows. *Physica D* **238**, 1507–1523 (2009).

[23] Froyland, G., Santitissadeekorn, N. & Monahan, A. Transport in time-dependent dynamical systems: Finite-time coherent sets. *Chaos* **20**, 043116–1–043116–16 (2010).

[24] Froyland, G., Gottwald, G. A. & Hammerlindl, A. A computational method to extract macroscopic variables and their dynamics in multiscale systems. *SIAM Journal on Applied Dynamical Systems* **13**, 1816–1846 (2014).

[25] Kaiser, E. *et al.* Cluster-based reduced-order modelling of a mixing layer. *J. Fluid Mech.* **754**, 365–414 (2014).

[26] Koopman, B. O. Hamiltonian systems and transformation in Hilbert space. *PNAS* **17**, 315–318 (1931). URL `http://www.pnas.org/content/17/5/315.short`. `http://www.pnas.org/content/17/5/315.full.pdf+html`.

[27] Koopman, B. O. & Neumann, J.-v. Dynamical systems of continuous spectra. *Proceedings of the National Academy of Sciences of the United States of America* **18**, 255 (1932).

[28] Mezić, I. Spectral properties of dynamical systems, model reduction and decompositions. *Nonlinear Dynamics* **41**, 309–325 (2005).

[29] Rowley, C. W., Mezić, I., Bagheri, S., Schlatter, P. & Henningson, D. Spectral analysis of nonlinear flows. *Journal of Fluid Mechanics* **645**, 115–127 (2009).

[30] Budišić, M. & Mezić, I. An approximate parametrization of the ergodic partition using time averaged observables. *48th IEEE Conf. on Decision and Control* 3162–3168 (2009).

[31] Budišić, M. & Mezić, I. Geometry of the ergodic quotient reveals coherent structures in flows. *Physica D* **241**, 1255–1269 (2012).

[32] Lan, Y. & Mezić, I. Linearization in the large of nonlinear systems and Koopman operator spectrum. *Physica D* **242**, 42–53 (2013).

[33] Mezić, I. Analysis of fluid flows via spectral properties of the Koopman operator. *Annual Review of Fluid Mechanics* **45**, 357–378 (2013).

[34] Gaspard, P., Nicolis, G. & Provata, A. Spectral signature of the pitchfork bifurcation: Liouville equation approach. *Physical Review E* **51**, 74–94 (1995).

[35] Bagheri, S. Koopman-mode decomposition of the cylinder wake. *Journal of Fluid Mechanics* **726**, 596–623 (2013).

[36] Brunton, S. L., Brunton, B. W., Proctor, J. L. & Kutz, J. Koopman observable subspaces and finite linear representations of nonlinear dynamical systems for control. *PLoS ONE* **11**, e0150171 (2016).

[37] Williams, M. O., Kevrekidis, I. G. & Rowley, C. W. A data-driven approximation of the Koopman operator: extending dynamic mode decomposition. *Journal of Nonlinear Science* (2015).

[38] Schmid, P. J. Dynamic mode decomposition of numerical and experimental data. *Journal of Fluid Mechanics* **656**, 5–28 (2010).

[39] Chen, K. K., Tu, J. H. & Rowley, C. W. Variants of dynamic mode decomposition: Boundary condition, Koopman, and Fourier analyses. *Journal of Nonlinear Science* **22**, 887–915 (2012).

[40] Tu, J. H., Rowley, C. W., Luchtenburg, D. M., Brunton, S. L. & Kutz, J. N. On dynamic mode decomposition: theory and applications. *Journal of Computational Dynamics* **1**, 391–421 (2014).

[41] Kutz, J. N., Brunton, S. L., Brunton, B. W. & Proctor, J. L. *Dynamic Mode Decomposition: Data-Driven Modeling of Complex Systems* (SIAM, 2016).

[42] Brunton, S. L., Proctor, J. L. & Kutz, J. N. Discovering governing equations from data by sparse identification of nonlinear dynamical systems. *PNAS* **113**, 3932–3937 (2016).

[43] Tibshirani, R. Regression shrinkage and selection via the lasso. *J. of the Royal Statistical Society B* 267–288 (1996).

[44] Wang, W. X., Yang, R., Lai, Y. C., Kovanis, V. & Grebogi, C. Predicting catastrophes in nonlinear dynamical systems by compressive sensing. *PRL* **106**, 154101 (2011).

[45] Koza, J. R., Bennett III, F. H. & Stiffelman, O. Genetic programming as a Darwinian invention machine. In *Genetic Programming*, 93–108 (Springer, 1999).

[46] Bongard, J. & Lipson, H. Automated reverse engineering of nonlinear dynamical systems. *Proceedings of the National Academy of Sciences* **104**, 9943–9948 (2007).

[47] Schmidt, M. & Lipson, H. Distilling free-form natural laws from experimental data. *Science* **324**, 81–85 (2009).

[48] Ozoliņš, V., Lai, R., Caflisch, R. & Osher, S. Compressed modes for variational problems in mathematics and physics. *Proceedings of the National Academy of Sciences* **110**, 18368–18373 (2013).

[49] Schaeffer, H., Caflisch, R., Hauck, C. D. & Osher, S. Sparse dynamics for partial differential equations. *Proceedings of the National Academy of Sciences USA* **110**, 6634–6639 (2013).

[50] Mackey, A., Schaeffer, H. & Osher, S. On the compressive spectral method. *Multiscale Modeling & Simulation* **12**, 1800–1827 (2014).

[51] Bai, Z. *et al.* Low-dimensional approach for reconstruction of airfoil data via compressive sensing. *AIAA Journal* 1–14 (2014).

[52] Proctor, J. L., Brunton, S. L., Brunton, B. W. & Kutz, J. N. Exploiting sparsity and equation-free architectures in complex systems (invited review). *The European Physical Journal Special Topics* **223**, 2665–2684 (2014).

[53] Brunton, S. L., Tu, J. H., Bright, I. & Kutz, J. N. Compressive sensing and low-rank libraries for classification of bifurcation regimes in nonlinear dynamical systems. *SIAM Journal on Applied Dynamical Systems* **13**, 1716–1732 (2014).

[54] Kevrekidis, I. G. *et al.* Equation-free, coarse-grained multiscale computation: Enabling microscopic simulators to perform system-level analysis. *Communications in Mathematical Science* **1**, 715–762 (2003).

[55] Kalman, R. E. A new approach to linear filtering and prediction problems. *Journal of Fluids Engineering* **82**, 35–45 (1960).

[56] Ho, B. L. & Kalman, R. E. Effective construction of linear state-variable models from input/output data. In *Proc. 3rd AAC*, 449–459 (1965).

[57] Welch, G. & Bishop, G. An introduction to the Kalman filter (1995).

[58] Dullerud, G. E. & Paganini, F. *A course in robust control theory: A convex approach*. Texts in Applied Mathematics (Springer, Berlin, Heidelberg, 2000).

[59] Skogestad, S. & Postlethwaite, I. *Multivariable feedback control: analysis and design* (John Wiley & Sons, Inc., Hoboken, New Jersey, 2005), 2 edn.

[60] Hermann, R. & Krener, A. J. Nonlinear controllability and observability. *IEEE Transactions on automatic control* **22**, 728–740 (1977).

[61] Aeyels, D. Generic observability of differentiable systems. *SIAM Journal on Control and Optimization* **19**, 595–603 (1981).

[62] Aguirre, L. A. & Letellier, C. Observability of multivariate differential embeddings. *Journal of Physics A: Mathematical and General* **38**, 6311 (2005).

[63] Farmer, J. D. & Sidorowich, J. J. Predicting chaotic time series. *Physical Review Letters* **59**, 845 (1987).

[64] Crutchfield, J. P. & McNamara, B. S. Equations of motion from a data series. *Complex Systems* **1**, 417–452 (1987).

[65] Sugihara, G. & May, R. M. Nonlinear forecasting as a way of distinguishing chaos from measurement error in time series. *Nature* **344**, 734–741 (1990).

[66] Rowlands, G. & Sprott, J. C. Extraction of dynamical equations from chaotic data. *Physica D* **58**, 251–259 (1992).

[67] Abarbanel, H. D. I., Brown, R., Sidorowich, J. J. & Tsimring, L. S. The analysis of observed chaotic data in physical systems. *Reviews of Modern Physics* **65**, 1331 (1993).

[68] Sugihara, G. *et al.* Detecting causality in complex ecosystems. *Science* **338**, 496–500 (2012).

[69] Ye, H. *et al.* Equation-free mechanistic ecosystem forecasting using empirical dynamic modeling. *PNAS* **112**, E1569–E1576 (2015).

[70] Grassberger, P. & Procaccia, I. Characterization of strange attractors. *Physical Review Letters* **50**, 346–349 (1983).

[71] Broomhead, D. S. & King, G. P. Extracting qualitative dynamics from experimental data. *Physica D: Nonlinear Phenomena* **20**, 217–236 (1986).

[72] Kaplan, D. T. & Glass, L. Direct test for determinism in a time series. *Physical Review Letters* **68**, 427–430 (1992).

[73] Juang, J. N. & Pappa, R. S. An eigensystem realization algorithm for modal parameter identification and model reduction. *J. of Guidance, Control, and Dynamics* **8**, 620–627 (1985).

[74] Broomhead, D. S. & Jones, R. Time-series analysis. *Proc. Roy. Soc. A* **423**, 103–121 (1989).

[75] Proctor, J. L., Brunton, S. L. & Kutz, J. N. Dynamic mode decomposition with control. *SIAM Journal on Applied Dynamical Systems* **15**, 142–161 (2016).

[76] Lorenz, E. N. Deterministic nonperiodic flow. *Journal of Atmospheric Sciences* **20**, 130–141 (1963).

[77] Cousins, W. & Sapsis, T. P. Quantification and prediction of extreme events in a one-dimensional nonlinear dispersive wave model. *Physica D* **280**, 48–58 (2014).

[78] Babaee, H. & Sapsis, T. P. A minimization principle for the description of modes associated with finite-time instabilities. *Proc. R. Soc. A* **472** (2016).

[79] Cousins, W. & Sapsis, T. P. Reduced-order precursors of rare events in unidirectional nonlinear water waves. *Journal of Fluid Mechanics* **790**, 368–388 (2016).

[80] Gavish, M. & Donoho, D. L. The optimal hard threshold for singular values is $4/\sqrt{3}$. *IEEE Transactions on Information Theory* **60**, 5040–5053 (2014).

[81] Cox, A., Doell, R. R. & Dalrymple, G. B. Reversals of the Earth's magnetic field. *Science* **144**, 1537–1543 (1964).

[82] Guyodo, Y. & Valet, J.-P. Global changes in intensity of the Earth's magnetic field during the past 800 kyr. *Nature* **399**, 249–252 (1999).

[83] Pétrélis, F. & Fauve, S. Chaotic dynamics of the magnetic field generated by dynamo action in a turbulent flow. *Journal of Physics: Condensed Matter* **20**, 494203 (2008).

[84] Pétrélis, F., Fauve, S., Dormy, E. & Valet, J.-P. Simple mechanism for reversals of Earth's magnetic field. *Physical Review Letters* **102**, 144503 (2009).

[85] Pétrélis, F. & Fauve, S. Mechanisms for magnetic field reversals. *Philosophical Transactions of the Royal Society of London A: Mathematical, Physical and Engineering Sciences* **368**, 1595–1605 (2010).

[86] Schreiber, T. & Kaplan, D. T. Nonlinear noise reduction for electrocardiograms. *Chaos: An Interdisciplinary Journal of Nonlinear Science* **6**, 87–92 (1996).

[87] Richter, M. & Schreiber, T. Phase space embedding of electrocardiograms. *Physical Review E* **58**, 6392 (1998).

[88] Keogh, E., Lin, J. & Fu, A. HOT SAX: Efficiently finding the most unusual time series subsequence. In *Fifth IEEE International Conference on Data Mining* (2005).

[89] Laguna, P., Mark, R. G., Goldberg, A. & Moody, G. B. A database for evaluation of algorithms for measurement of qt and other waveform intervals in the ecg. In *Computers in Cardiology 1997*, 673–676 (IEEE, 1997).

[90] Goldberger, A. L. *et al.* Physiobank, physiotoolkit, and physionet components of a new research resource for complex physiologic signals. *Circulation* **101**, e215–e220 (2000).

[91] Pritchard, W. S. & Duke, D. W. Measuring chaos in the brain: a tutorial review of nonlinear dynamical EEG analysis. *International Journal of Neuroscience* **67**, 31–80 (1992).

[92] Acharya, R., Faust, O., Kannathal, N., Chua, T. & Laxminarayan, S. Non-linear analysis of EEG signals at various sleep stages. *Computer methods and programs in biomedicine* **80**, 37–45 (2005).

[93] Kannathal, N., Choo, M. L., Acharya, U. R. & Sadasivan, P. K. Entropies for detection of epilepsy in EEG. *Computer methods and programs in biomedicine* **80**, 187–194 (2005).

[94] Stam, C. J. Nonlinear dynamical analysis of EEG and MEG: review of an emerging field. *Clinical Neurophysiology* **116**, 2266–2301 (2005).

[95] Lainscsek, C., Hernandez, M. E., Poizner, H. & Sejnowski, T. J. Delay differential analysis of electroencephalographic data. *Neural computation* (2015).

[96] Kemp, B., Zwinderman, A. H., Tuk, B., Kamphuisen, H. A. & Oberye, J. J. Analysis of a sleep-dependent neuronal feedback loop: the slow-wave microcontinuity of the eeg. *IEEE Transactions on Biomedical Engineering* **47**, 1185–1194 (2000).

[97] Schaffer, W. M. & Kot, M. Do strange attractors govern ecological systems? *BioScience* **35**, 342–350 (1985).

[98] London, W. P. & Yorke, J. A. Recurrent outbreaks of measles, chickenpox and mumps i. seasonal variation in contact rates. *American journal of epidemiology* **98**, 453–468 (1973).
